# Cell Type‐Specific Expression of p16, p21, and p53 Reveals Age‐Dependent Glial Senescence in the *App^NL‐G‐F^* Mouse Model of Alzheimer's Disease

**DOI:** 10.1111/acel.70478

**Published:** 2026-04-14

**Authors:** Eileen Mac Sweeney, Giulia Abate, Bjorn R. V. Bakker, Andrea Mastinu, Ylenia Lai, Daniela Uberti, Per Nilsson, Simone Tambaro

**Affiliations:** ^1^ Division of Neurogeriatrics, Department of Neurobiology, Care Sciences and Society Karolinska Institutet Solna Sweden; ^2^ Division of Pharmacology, Department of Molecular and Translational Medicine University of Brescia Brescia Italy; ^3^ Department of Biological Sciences University of Cagliari, Cittadella Universitaria di Monserrato Monserrato Italy

**Keywords:** Alzheimer's disease, *App* ^*NL‐G‐F*^, p16, p21, p53, senescence

## Abstract

Cellular senescence, a state of irreversible cell cycle arrest, plays a key role in neurodegenerative diseases, including Alzheimer's disease (AD). While senescent cells are emerging as potential therapeutic targets, the dynamics of their occurrence over time and the specific cell types most affected by AD are still not well understood. This study investigates age‐ and pathology‐dependent changes in senescence markers, specifically p16, p21, and p53, using the amyloidogenic *App* ^*NL‐G‐F*^ knock‐in AD mouse model. Female *App* ^*NL‐G‐F*^ and wild‐type (WT) mice were evaluated at 4, 12, and 24 months of age to capture disease progression changes from early to advanced AD stages. Immunofluorescence and qPCR were used to quantify p16, p21, and p53 expressions. Senescence‐associated β‐galactosidase (SA‐β‐Gal) activity, IL‐1β and IL‐6 levels, and CD68‐p21 colocalization were assessed. At 4 months‐of‐age, only p21 levels in astrocytes differ between genotypes. By 12 months, *App* ^*NL‐G‐F*^ mice exhibited increased p16 and p21 expression in glial cells, along with elevated SA‐β‐Gal activity. IL‐1β level increased in the cortex and hippocampus, while IL‐6 only in the hippocampus. Most CD68‐positive microglia co‐expressed p21 in both hippocampus (73%) and cortex (82%), indicating a prevalent senescent phenotype among reactive microglia. p16 and p21 changes became more pronounced at 24 months. p53 expression followed a distinct pattern, increasing in astrocytes at 12 months and in microglia by 24 months. Neurons showed no genotype‐dependent differences. These findings reveal a progressive, amyloid‐linked glial senescence response, supporting the *App* ^*NL‐G‐F*^ model as a reliable platform for evaluating senotherapeutic strategies in AD.

## Graphical abstract

Longitudinal senescence profiling in *App* ^*NL‐G‐F*^ Alzheimer's mice reveals progressive microglial and astrocytic senescence (p16/p21 upregulation, morphological impairment, SA‐β‐galactosidase activity, and elevated IL‐1β and IL‐6) without neuronal senescence. Amyloid pathology is specifically linked to glial senescence, suggesting these cells as primary targets for senotherapeutic intervention in Alzheimer's disease.

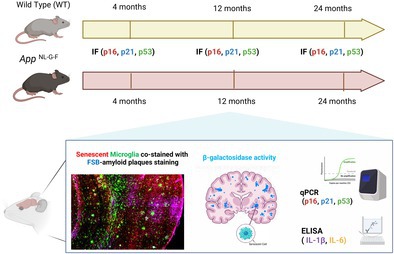

## 1 Introduction

Senescent cells, often referred to as “zombie cells”, are characterized by an irreversible state of cell cycle arrest and various biological events, including telomere shortening, DNA damage, oxidative stress, mitochondrial dysfunction, and oncogene activation (Gorgoulis et al. 2019; Scudellari 2017). Initially identified as a tumor‐suppressive mechanism to prevent the proliferation of damaged or potentially cancerous cells, senescence also plays critical roles in processes such as tissue remodeling during development and wound healing (Jun and Lau 2010; Storer et al. 2013).

The growth arrest observed in senescent cells is associated with an upregulation of the tumor suppressors p16 and p21. p16 is a well‐characterized cyclin‐dependent kinase inhibitor that plays a crucial role in inducing and maintaining cell cycle arrest, and its expression is known to increase with age in various tissues, including the brain, and has been consistently observed in neurodegenerative diseases (Graves et al. 2025; Takeuchi et al. 2010). Similarly, p21 is another cyclin‐dependent kinase inhibitor involved in cell cycle regulation and in the establishment of senescence, often acting downstream of the tumor suppressor protein p53, which is involved in regulates several genes that play crucial roles in arresting the cell cycle and repairing damaged DNA (Takeuchi et al. 2010). Numerous studies have demonstrated that p53 is vital for protecting the genome in response to DNA damage (Hill et al. 2024; Wang et al. 2022; Zhang et al. 2024). The senescence process may or may not involve the activation of p53 (Passos et al. 2010). In senescent cells, the activation of p21 and p53 can be temporary, as their protein levels generally decline once growth arrest is firmly established (De Cecco et al. 2019; Stein et al. 1999; Sturmlechner et al. 2022). While p21 expression might decrease after its initial peak, p16 expression may increase, contributing to the maintenance of growth arrest in certain, though not all, senescent cells (Stein et al. 1999). Senescent cells exhibit characteristic phenotypic changes, including increased cellular volume and heightened lysosomal activity, which are prominently detected by the expression of senescence‐associated β‐galactosidase (SA‐β‐gal) (Kurz et al. 2000). Telomere shortening and nuclear alterations also emerge as hallmarks of cellular senescence, including persistent nuclear localization of DNA damage response proteins, such as reduced levels of the nuclear lamina protein Lamin B1 (LMNB1) (Baker and Petersen 2018; Ogrodnik et al. 2024). Despite these protective roles, senescent cells can have a harmful impact on surrounding homeostasis, particularly in the context of aging and chronic diseases (Tominaga 2015). Recent evidence has further revealed that senescence can propagate between brain immune cells through paracrine mechanisms during aging, whereby senescent‐like border‐associated macrophages transfer senescence‐associated signals to neighboring microglia via migrasomes, contributing to microglial dysfunction and cognitive decline (Hu et al. 2025).

Furthermore, senescent cells secrete a pro‐inflammatory cocktail of cytokines, chemokines, growth factors, and proteases collectively referred to as the senescence‐associated secretory phenotype (SASP) (Coppé et al. 2010), which are involved in various processes such as tissue repair, while chronic secretion of SASP results in tissue damage and cell death (Schafer et al. 2020).

The accumulation of senescent cells has been observed in the pathophysiology of various age‐related diseases, including cardiovascular diseases, diabetes, and neurodegenerative disorders (Gardner et al. 2015; Kitada et al. 2014; Martínez‐Cué and Rueda 2020; Wang et al. 2015). Senescence in the central nervous system (CNS) is associated with several neurodegenerative diseases, such as Alzheimer's disease (AD), Parkinson's disease, and Huntington's disease. In AD, emerging evidence suggests that senescent glial cells contribute significantly to the onset of the pathology (Hudson et al. 2025). In the brain, cellular senescence affects both dividing cells, such as glia (including astrocytes and microglia), and post‐mitotic cells, such as neurons (Herdy et al. 2022). Microglia, the resident immune cells of the brain, undergo a senescence‐like state characterized by impaired phagocytic activity and an increased pro‐inflammatory SASP, which can exacerbate amyloid plaque deposition (Hudson et al. 2025). Notably, single‐nucleus transcriptomic analysis of human postmortem AD brains has identified premature senescence predominantly in glial cells, particularly microglia (Fancy et al. 2024). Similarly, senescent astrocytes lose their neuroprotective functions, contributing to synaptic dysfunction and neuronal degeneration (Ungerleider et al. 2021). Neurons can also exhibit features of senescence or “neurescence,” including DNA damage and the activation of cell cycle regulators like p16 and p21 (Hudson et al. 2025). Neurescence can be associated with a loss of neuronal identity, including reduced expression of canonical neuronal genes, simplified neuritic arborization, and the retraction or loss of dendritic spines (Chou et al. 2023).

The removal of senescent cells is an emerging promising therapeutic strategy to target the aging process and help prevent or reduce aging‐related diseases, such as AD (Riessland and Orr 2023; Zhang et al. 2019). This therapeutic strategy comprises compounds that eliminate senescent cells (senolytics) or modulate SASP (senomorphics) to reduce neuroinflammation and restore tissue homeostasis (Zhang et al. 2019). However, for these therapeutic strategies to be effective, appropriate pre‐clinical models of senescence related to AD with high translational value are required.

Furthermore, a significant gap remains in understanding the interplay between senescence and AD pathology. AD is primarily characterized by the early and abnormal accumulation of amyloid‐beta (Aβ) peptides, which leads to the formation of toxic oligomers and plaques. This initiates a pathological cascade that ultimately results in the formation of neurofibrillary tangles composed of hyperphosphorylated Tau proteins (Roda et al. 2022). This cascade contributes to widespread synaptic dysfunction, neuroinflammation, and neurodegeneration. While a link between tauopathy and senescence induction has been demonstrated in vivo, the effect of Aβ pathology on this process remains unclear (Dorigatti et al. 2022; Musi et al. 2018). Furthermore, the precise involvement of different brain cell types in the progression of amyloid‐induced senescence is poorly investigated. Addressing these knowledge gaps is critical, as it could clarify the contribution of different brain cell types to disease progression and facilitate the design of effective senescence‐targeting therapies for AD.

Taking this into consideration, this study aimed to investigate the alterations in senescence markers across different stages of disease progression in the amyloidogenic amyloid precursor protein (*App*) knock‐in AD mouse model *App* ^*NL‐G‐F*^, focusing on the expression of key senescence‐associated markers p16, p21, and p53 in neurons, microglia, and astrocytes. The *App* ^*NL‐G‐F*^ mice carry a humanized Aβ42 sequence and feature the Swedish (NL), Iberian (F), and Arctic (G) mutations in the amyloid precursor protein (APP). These mutations lead to significant Aβ pathology, gliosis, and memory impairment, starting at 7 months of age, making them a widely accepted model for studying AD‐related mechanisms (Saito et al. 2014). We examined the expression levels of p16, p21, and p53, at early (4 months), advanced (12 months), and late (20–24 months) stages of Aβ pathology. Additionally, we investigated lysosomal dysfunction markers such as β‐galactosidase and pro‐inflammatory cytokines, including interleukin‐1β (IL‐1β) and interleukin‐6 (IL‐6). We also assessed the distribution and morphology of senescent microglia and the colocalization with reactive microglia.

Our results show the accumulation of senescent glial cells in the *App* ^*NL‐G‐F*^ mouse model of AD and reveal distinct, stage‐dependent changes in senescence‐associated markers. Notably, we did not detect any pathology‐related senescence in neurons. These findings emphasize the potential of the *App* ^*NL‐G‐F*^ mouse model for targeting glial senescence as a therapeutic strategy to counteract Aβ‐driven pathology in AD.

## 2 Materials and Methods

### 2.1 Mouse Models

All mice used in this study were bred in the animal facility Comparative Medicine Biomedicum (KM‐B), Solna campus, Karolinska Institutet. Female *App* ^*NL‐G‐F*^ mice (Apptm3.1Tcs/Apptm3.1Tcs) and C57BL/6J mice of the same genetic background were used as controls. Mice were housed in groups of three to five individuals, and the light–dark cycle was 12‐h:12‐h (lights on at 7:00). Ethical permission for the described animal experiments was obtained from the Stockholm animal ethical board (15758–2019 and 12570–2021). Animal housing and care were conducted under the supervision of licensed veterinarians, with technical support provided by trained personnel. All measures were taken to minimize the use of animals in accordance with ethical guidelines. Female Wilde Type (WT) and *App* ^*NL‐G‐F*^ mice aged 4, 12, and 22/24 months were anesthetized with 2% isoflurane and subjected to intracardiac perfusion with PBS to remove blood. For each experimental group, 3–5 mice were used. Brains were promptly extracted and divided into two hemispheres. One hemisphere was fixed in 10% formalin for subsequent morphological and immunohistochemical analyses, while the other hemisphere was dissected to isolate the hippocampus and cortex for biochemical investigations.

### 2.2 Immunofluorescence Analysis

Brain tissue sections (5 μm) of WT and *App* ^*NL‐G‐F*^ mice at 4, 12, and 24 months of age were put on glass slides and deparaffinized by washing in Xylene and decreasing concentrations of ethanol (99%–70%). For antigen retrieval, slides were pressure‐boiled in a citrate buffer solution (0.1 M citric acid and 0.1 M sodium citrate) at 110°C for 5 min, then washed with tap water, followed by 3 washes in Phosphate‐Buffered Saline, Tween 0.05% (PBS‐T) for 5 min each. Sections were then incubated with normal goat serum in PBS‐T (Vector Laboratories, USA) for 30 min at room temperature. Samples were washed 3× in PBS‐T for 5 min each with slow agitation, then incubated with the primary antibody at 4°C overnight (Supplementary Table S1). Thereafter, sections were washed 3× in PBS‐T for 5 min each with slow agitation and incubated with secondary antibodies (Table S1) for 2 h at room temperature. After the washing step, samples were incubated for 15 min with slow agitation with Hoechst solution, 1:500 in PBS‐T. For amyloid plaque visualization, brain sections were incubated with FSB (1‐Fluoro‐2,5‐bis(3‐carboxy‐4‐hydroxystyryl)benzene) for 30 min. After the samples were washed 3× in PBS‐T for 5 min each, followed by mounting with PermaFluor Aqueous Mounting Medium (ThermoScientific, USA), and kept for drying overnight. Samples were analyzed with a Zeiss AXIO Observer.Z1 Inverted Fluorescence Microscope (Carl Zeiss, Jena, Germany) in 4×, 10×, and 20× magnifications and image reconstruction was performed with an LSM Zen Blue Image Examiner (Carl Zeiss S.*p*.A., Milan, Italy). Quantification of immunoreactive signals was performed using ImageJ (National Institutes of Health, MD) by counting the total number of neuronal or glial cells positive for the senescence markers in the hippocampus and retrosplenial cortex, and normalizing to the total number of cells. Data are expressed as the percentage of marker‐positive cells relative to the total cell population, allowing normalized comparison across conditions, or as total cell counts normalized per tissue area to assess the absolute senescent cell burden.

### 2.3 Beta‐Galactosidase Staining

SA‐β‐gal staining was performed on brain sections of WT and *App* ^*NL‐G‐F*^ mouse tissues at 12 months of age. After perfusion with PBS, the mouse brains were removed, embedded with OCT solution and immediately frozen in cold 2‐methylbutane for cryopreservation; tissues were then stored at −80°C. The brains were sectioned using a cryostat (Leica CM1860) with 10 μm section thickness. SA‐β‐gal staining was performed according to the protocol of the Senescence‐β‐Galactosidase Staining Kit (#9860, Cell Signaling Technology, Danvers, MA, USA). For the double staining with Iba1, tissues were further incubated overnight with the primary antibody and 2 h at room temperature with the secondary antibody. Images were acquired with a Nikon Eclipse E800 light microscope in 10× and 20× magnifications. Quantification was performed using ImageJ software by determining the percentage of SA‐β‐gal positivity per ROI.

### 2.4 RNA Isolation, and qPCR

RNA was extracted from hippocampus and cortex of WT and *App* ^*NL‐G‐F*^ mouse tissues at 12 months of age using RNeasy Mini Kit (74,104, Qiagen), according to the manufacturer's instructions. RNA quality (RNA integrity number) and quantity were measured by NanoDrop ND1000 Spectrophotometer (ThermoFisher). 1 μg of RNA per sample was reverse‐transcribed into cDNA. The RNA was mixed with 20 μL of master mix and amplified by using a cycler (S1000 Thermal cycler Bio‐Rad). For the RT‐qPCR a total of 10 μL reactions were run in duplicates using TaqMan Fast advanced Mastermix (ThermoFisher, Waltham, MA, USA) and StepOne Plus real‐time PCR Detection System (Applied Biosystem, Waltham, MA, USA). The primers (ThermoFisher) used in this study were: p16 (Mm0049449_m1), p21 (Mm00432448_m1), p53 (Mm01731290_g1), IL‐1β(Mm00434228_m1), IL‐6 (Mm00446190_m1), and β‐Actin (Mm02619580_g1). The expression levels were normalized against β‐Actin. Relative quantification (RQ) for mRNA was calculated using the ΔΔ cycle threshold (ΔΔCT) method, with fold changes using the formula, ΔΔCt = ΔCtT2‐ΔCtT1.

### 2.5 Elisa

IL‐1β and IL‐6 protein levels in the hippocampus and cortex of 12‐month‐old WT and *App* ^*NL‐G‐F*^ mice were determined using enzyme‐linked immunosorbent assays (ELISAs), according to the manufacturer's instructions (DuoSet kits, R&D Systems, Minneapolis, USA). The results were expressed in pg/mL.

### 2.6 Skeletal and Fractal Analysis

Morphological changes in microglia were analyzed using skeletal and fractal analyses, following the method established by Young and Morrison on hippocampus and cortex microglia of 12‐month‐old WT and *App* ^*NL‐G‐F*^ mice. Images at 20× were analyzed using ImageJ software, with AnalyzeSkeleton (2D/3D) and FracLac plugins. Specifically, for the Skeletal analysis, three mice per genotype were used, and cells Iba1^+^ were analyzed. The number of microglia endpoints/cell and microglia process length/cell were then calculated. For the Fractal analysis, four mice per genotype were analyzed, considering 10 cells p21^+^ and 10 cells p21^−^ per mouse. Fractal dimension and lacunarity were then calculated.

### 2.7 Statistical Analysis

Data were expressed as mean ± standard error of the mean (SEM) and analyzed with the GraphPad Prism 10 software (GraphPad Software Inc., CA, USA). Results were analyzed by multiple *t*‐tests or an unpaired Student's *t*‐test. For comparisons among more than two groups, one‐way ANOVA or two‐way ANOVA test was used, followed by Tukey's multiple comparison test. Variability of the estimates was reported as the standard error of the mean (SEM). A heatmap was generated in GraphPad Prism to visualize the age‐dependent changes between genotypes and cell types.

## 3 Results

### 3.1 Cell‐Type–Specific Analysis Reveals Early Microglial Senescence in *App* ^*NL‐G‐F*^ Mice

**FIGURE 1:**
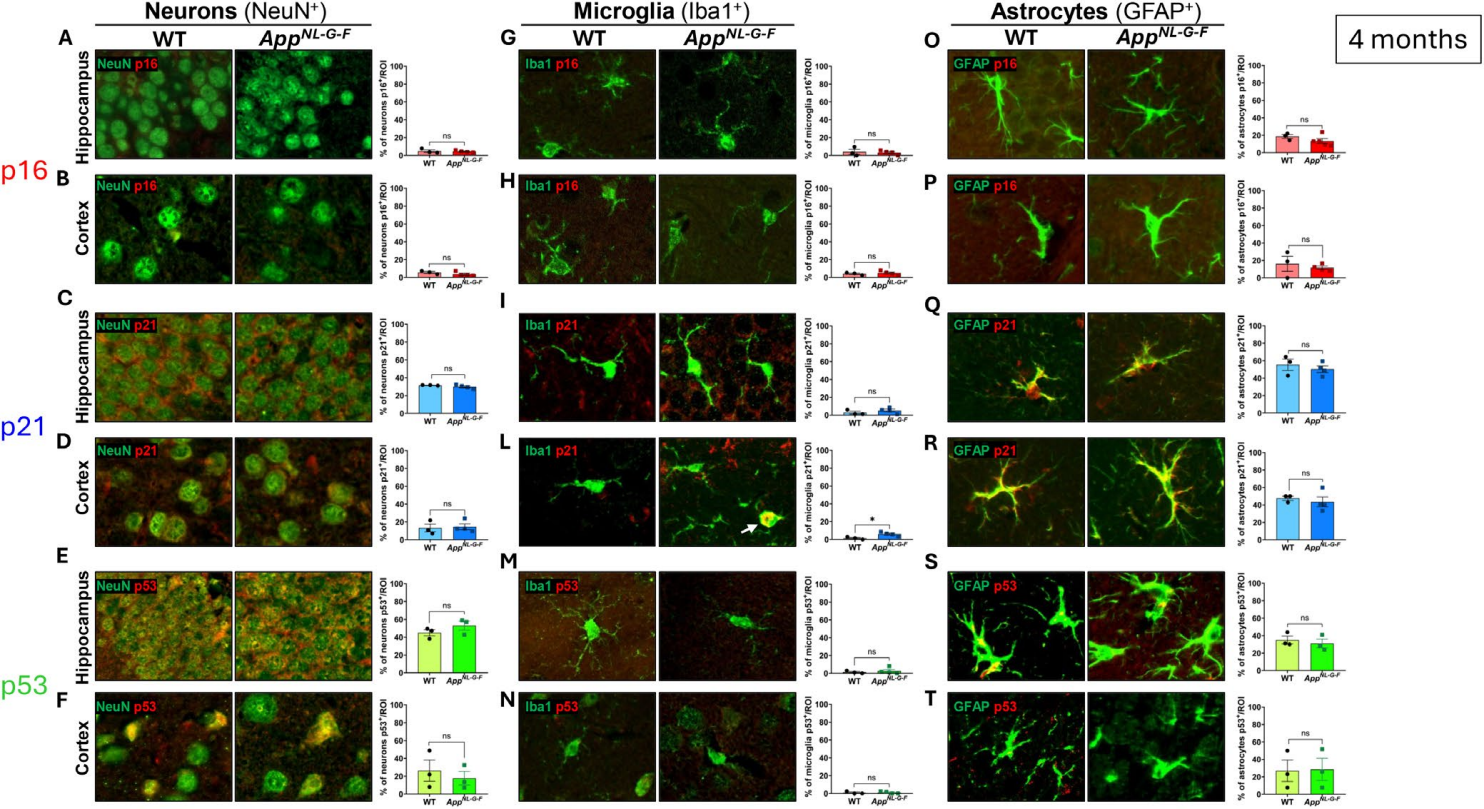
Cell‐specific immunofluorescence analysis of senescence markers p16, p21, and p53 at 4 months of age. Immunofluorescence co‐labeling with neuronal (NeuN), microglial (Iba1), and astrocytic (GFAP) markers was performed to evaluate cellular senescence status. Quantification of NeuN^+^ (A–F), Iba1^+^ (G–N), GFAP^+^ (O–T) cells co‐expressing p16, p21, and p53 in the hippocampus and cortex of WT (*n* = 3) and *App* ^*NL‐G‐F*^ (*n* = 4) mice at 4 months of age. (L) A significant increase in p21^+^ microglial cells was observed in the cortex of *App* ^*NL‐G‐F*^ mice compared to WT. Scale bars: 50 μm. Data are presented as mean ± SEM. Statistical analysis: Unpaired *t*‐test. *p < 0.05 WT group.

To investigate cellular senescence in the context of both normal aging and AD‐like pathology, we analyzed the expression of senescence markers in female WT and *App* ^*NL‐G‐F*^ mice at 4, 12, and 24 months of age. We first focused on the early stage of pathology by examining 4‐month‐old *App* ^*NL‐G‐F*^ mice, a time point at which initial Aβ plaque deposition has been previously reported (Saito et al. 2014). To determine the cellular localization of senescence markers, we performed co‐immunostaining for p16, p21, and p53, together with cell‐type–specific markers for neurons (NeuN), microglia (Iba1), and astrocytes (GFAP) (Figure 1). No significant differences were observed between WT and *App* ^*NL‐G‐F*^ mice in the percentage of neuronal, microglial, or astrocytic cells expressing p16 or p53 in the hippocampus nor the cortex (*p* < 0.05) (Figure 1, Table S2). However, despite the overall low abundance of p21^+^ (Table S2), a significant increase of the percentage of p21^+^ microglial cells was detected in the cortex of *App* ^*NL‐G‐F*^ (5.1%) mice compared to WT (2.8%) controls (*F*(3,2) = 1.99, *p* = 0.025) (Figure 1L), suggesting an early onset of microglial senescence associated with AD‐like pathology. In contrast, p21 expression levels did not differ in hippocampal microglia or in neurons and astrocytes located in either the hippocampus or cortex (*p* < 0.05).

### 3.2 Glial Cells Exhibit Pronounced Senescence in 12‐Month‐Old *App* ^*NL*^ ^*‐G‐F*^ Mice With AD‐Like Pathology

By 12 months of age, pathology in *App* ^*NL‐G‐F*^ mice has markedly progressed, with extensive amyloid plaque accumulation in both the cortex and hippocampus, accompanied by pronounced microgliosis and astrogliosis (Saito et al. 2014). To assess whether these pathological alterations are associated with cellular senescence, we next analyzed the expression and cell‐type distribution of the senescence markers p16, p21, and p53 by immunofluorescence, using NeuN, Iba1, and GFAP to identify neurons, microglia, and astrocytes, respectively.

**FIGURE 2:**
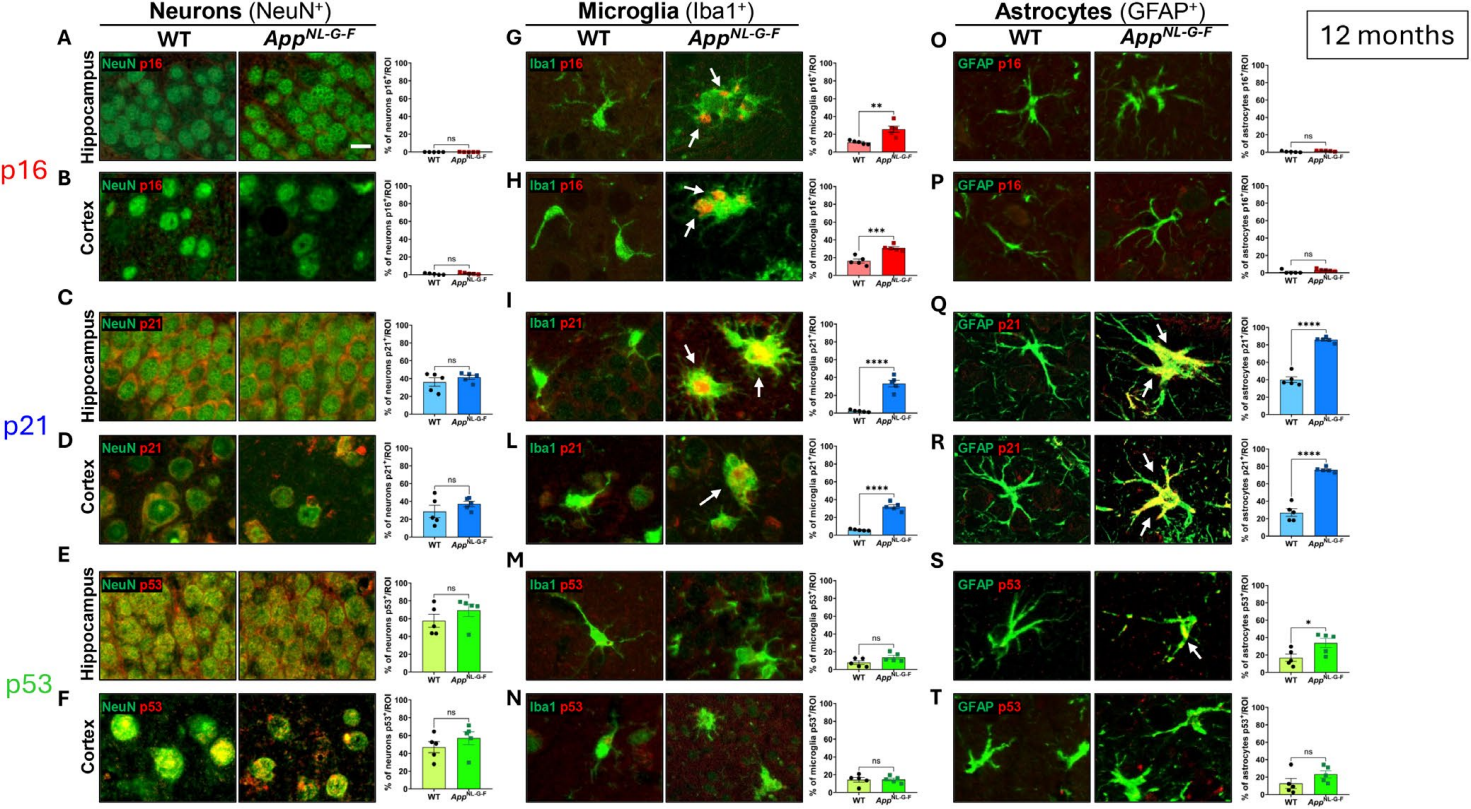
Cell‐specific immunofluorescence analysis of senescence markers p16, p21, and p53 at 12 months of age. Immunofluorescence co‐labeling with neuronal (NeuN), microglial (Iba1), and astrocytic (GFAP) markers was performed to evaluate cellular senescence status. Quantification of NeuN^+^ (A–F), Iba1^+^ (G–N), GFAP^+^ (O–T) cells co‐expressing p16, p21, and p53 in the hippocampus and cortex of WT (*n* = 5) and *App* ^*NL‐G‐F*^ (*n* = 5) mice at 12 months of age. Significant increases in microglia positive for p16 (G‐H) and microglia and astrocytes positive for p21 (I–L, Q, R) were observed in *App* ^*NL‐G‐F*^ mice. p53 was elevated in GFAP^+^ astrocytes in the hippocampus only (S). Scale bars: 50 μm. Data are presented as mean ± SEM. Statistical analysis: Unpaired *t*‐test. *p < 0.05, **p < 0.01, ***p < 0.001; ****p < 0.0001 versus WT group.

In neurons, p16 immunoreactivity was detected only in a small percentage of cells, and its expression did not differ between WT (0.02% in hippocampus; 0.8% in cortex) and *App* ^*NL‐G‐F*^ mice (0.06% in hippocampus; 1.5% in cortex) (*F*_hippo_(4,4) = 5.29, *p* = 0.616; *F*_cortex_(4,4) = 1.82, *p* = 0.317) (Figure 2A,B, Table S2). Similarly, although p21 and p53 were present in higher levels in neurons (for p21 35.9% in WT hippocampus, 41.3% in *App* ^*NL‐G‐F*^ hippocampus, 28.6% in WT cortex and 37.1% in *App* ^*NL‐G‐F*^ cortex; for p53 57.5% in WT hippocampus, 69.3% in *App* ^*NL‐G‐F*^ hippocampus, 46.9% in WT cortex and 57.0% in *App* ^*NL‐G‐F*^ cortex), no genotype‐dependent differences were observed in the hippocampus nor in the cortex (for p21 *F*_hippo_(4,4) = 3.98, *p* = 0.342; *F*_cortex_(4,4) = 5.25, *p* = 0.297; for p53 *F*_hippo_(4,4) = 1.46, *p* = 0.278; *F*_cortex_(4,4) = 1.32, *p* = 0.320) (Figure 2C–F, Table S2). These findings suggest that, at this stage, neuronal p16, p21, and p53 levels are not affected by the presence of amyloid pathology. In contrast, microglial cells exhibited a clear senescence‐associated profile. The percentage of p16^+^ and p21^+^ microglia was significantly higher in both the hippocampus (28.8% for p16, 36.0% for p21) and cortex (30.8% for p16, 33.4% for p21) of *App* ^*NL‐G‐F*^ mice compared to the hippocampus (11.0% for p16, 2.4% for p21) and cortex (16.3% for p16, 5.6% for p21) of WT controls (for p16: *F*_hippo_(4,4) = 16.63, *p* = 0.004; *F*_cortex_(4,4) = 2.10, *p* = 0.0006; for p21: *F*_hippo_(2,2) = 19.62, *p* = 0.001; *F*_cortex_(2,2) = 36.49, *p* = 0.0005) (Figure 2G–L), indicating that microglial senescence develops alongside amyloid pathology and neuroinflammation. Notably, p53 expression remained unchanged between groups (*F*_hippo_(4,4) = 1.07, *p* = 0.082; *F*_cortex_(4,4) = 2.65, *p* = 0.922) (Figure 2M,N), suggesting that p16/p21 pathways, rather than p53, may predominantly drive microglial senescence in this context. Among astrocytes, p16 expression remained very low and not significantly different across genotypes (in hippocampus 1.22% in WT, and 2.04% in *App* ^*NL‐G‐F*^, *F*_hippo_(4,4) = 3.45, *p* = 0.124; in cortex 0.93% in WT and 3.04% in *App* ^*NL‐G‐F*^; *F*_cortex_(4,4) = 1.19, *p* = 0.132) (Figure 2O,P), whereas p21 positivity was markedly increased, rising in WT from 40.0% in hippocampus and 26.9% in cortex, in *App* ^*NL‐G‐F*^ mice 85.8% in hippocampus and 76.0% in cortex (*F*_hippo_(4,4) = 6.32, *p* = 0.0001; *F*_cortex_(4,4) = 1.19, *p* = 0.132) (Figure 2Q,R). This robust induction of p21 suggests that astrocytes undergo strong senescence‐like changes in response to chronic amyloid burden and gliosis. Interestingly, the percentage of p53^+^ astrocytes in *App* ^*NL‐G‐F*^ mice was also significantly different in the hippocampus (*F*(4,4) = 1.57, *p* = 0.036) but not in the cortex (*F*(4,4) = 1.89, *p* = 0.170) (Figure 2S,T), indicating region‐specific susceptibility of astrocytic populations to senescence signaling under AD‐like conditions.

### 3.3 Sustained Microglial and Astrocytic Senescence in 24‐Month‐Old *App* ^*NL‐G‐F*^ Mice

**FIGURE 3:**
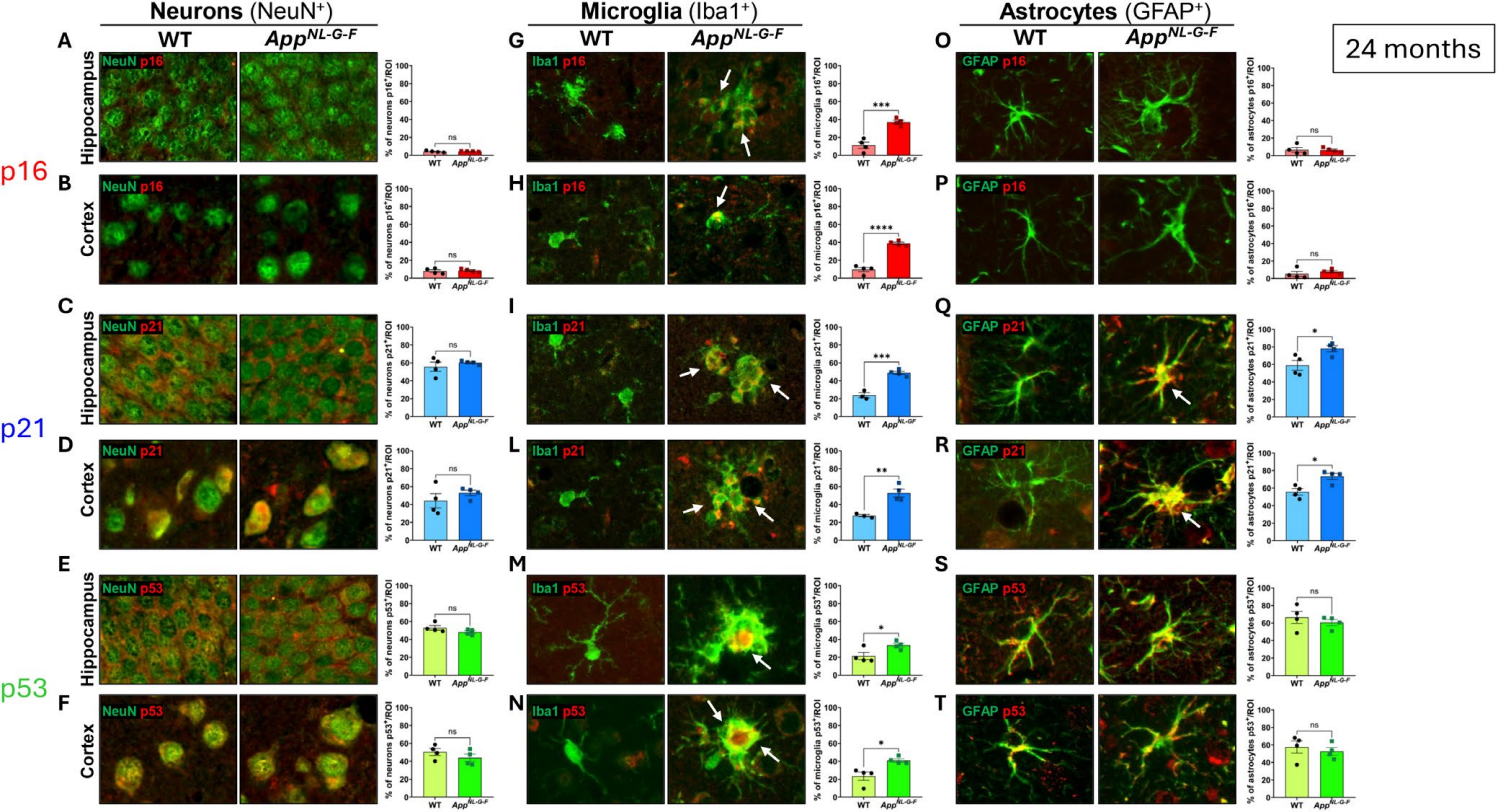
Cell‐specific immunofluorescence analysis of senescence markers p16, p21, and p53 at 24 months of age. Immunofluorescence co‐labeling with neuronal (NeuN), microglial (Iba1), and astrocytic (GFAP) markers was performed to evaluate cellular senescence status. Quantification of NeuN^+^ (A–F), Iba1^+^ (G–N), GFAP^+^ (O–T) cells co‐expressing p16, p21 and p53 in the hippocampus and cortex of WT (*n* = 4) and *App* ^*NL‐G‐F*^ (*n* = 4) mice at 24 months of age. Significant increases in p16 (G‐H), p21 (I‐L) and p53 (M‐N) in microglia and p21 (Q, R) in astrocytes of *App* ^*NL‐G‐F*^ mice. Scale bars: 50 μm. Data are presented as mean ± SEM (*n* = 4–5 per group). Statistical analysis: Unpaired *t*‐test. *p < 0.05, **p < 0.01, ***p < 0.001; ****p < 0.0001 versus WT group.

The presence of a senescence state in *App* ^*NL‐G‐F*^ mice at 24 months of age was also assessed. Immunofluorescence analysis, as previously conducted at 4 and 12‐months of age, showed no differences in p16^+^ neural cells in either the hippocampus (*F*(3,3) = 10.34, *p* = 0.815) (Figure 3A) nor cortex (*F*(3,3) = 2.49, *p* = 0.170) (Figure 3B) of *App* ^*NL‐G‐F*^ mice compared to WT mice, with both groups showing low marker positivity, below 10% of neurons (Table S2). Data showed that p21 (Figure 3C,D) and p53 (Figure 3E,F) were expressed in approximately 50% of neurons (Table S2), however, their expression levels did not differ significantly between genotypes (for p21: *F*_hippo_(3,3) = 25.74, *p* = 0.402; *F*_cortex_(3,3) = 6.59, *p* = 0.343; for p53: *F*_hippo_(3,3) = 2.38, *p* = 0.158; *F*_cortex_(3,3) = 1.123, *p* = 0.296). In microglia, all three senescence markers, p16, p21, and p53, were significantly higher in *App* ^*NL‐G‐F*^ mice compared to WT (for p16: *F*_hippo_(3,3) = 2.74, *p* = 0.0007; *F*_cortex_(3,3) = 2.81, *p* = 0.0001; for p21: *F*_hippo_(2,3) = 1.84, *p* = 0.0005; *F*_cortex_(2,3) = 15.25, *p* = 0.0054; for p53: *F*_hippo_(3,3) = 2.38, *p* = 0.158; *F*_cortex_(3,3) = 1.123, *p* = 0.296) (Figure 3G–N). In astrocytes, the percentage of p21^+^ cells was significantly higher in both the cortex (*F*(3,3) = 1.08, *p* = 0.011) and hippocampus (*F*(3,3) = 2.62, *p* = 0.027) of *App* ^*NL‐G‐F mice*^ compared to WT mice (Figure 3Q,R), while no significant differences were found in the percentage of p16^+^ (*F*_hippo_(2,3) = 4.60, *p* = 0.920; *F*_cortex_(3,3) = 4.52, *p* = 0.416) (Figure 3O,P) and p53^+^ (*F*_hippo_(3,3) = 3.80, *p* = 0.477; *F*_cortex_(3,3) = 2.48, *p* = 0.575) (Figure 3S,T) astrocytes between the two genotypes.

Summarizing the immunoexpression data across the three markers shows that the pathology in *App* ^*NL‐G‐F*^ mice causes a severe, age‐dependent cellular response, mainly characterized by the accumulation of positive microglia and astrocytes (Figure S1A). This strongly indicates that glial senescence and/or cell‐cycle‐related stress mechanisms are key components of the pathology in this model. Neurons, although positive for senescent markers, exhibit less noticeable changes compared to the significant activation observed in microglia and astrocytes.

### 3.4 Total Number Per Area of p16, p21, and p53‐Positive Neurons, Microglia, and Astrocytes in the Hippocampus and Cortex of WT and *App* ^*NL‐G‐F*^ Mice Across Aging

To complement our previous analysis, we next performed a quantitative assessment of the total number of p16‐, p21‐, and p53‐positive neurons, microglia, and astrocytes per defined area in both the hippocampus and cortex in WT and *App* ^*NL‐G‐F*^ mice across the examined ages (4, 12, and 24 months of age) to capture age‐dependent changes across brain regions and genotypes. By reporting the total cell counts normalized per tissue area, we aimed to evaluate the accumulation of senescent cells and their potential contribution to AD progression and aging‐related neurobiology.

**FIGURE 4:**
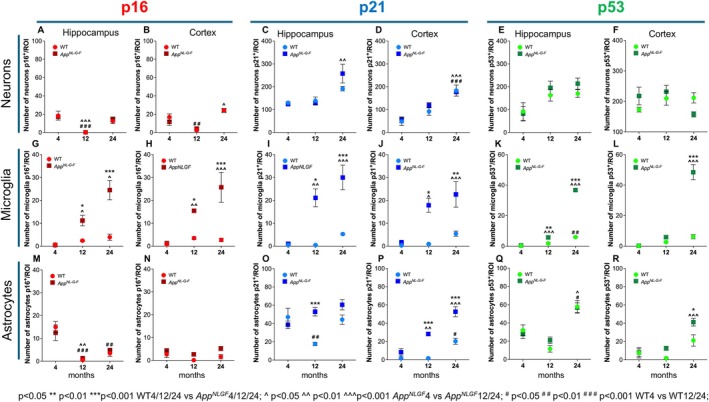
Count of cells positive to senescence markers across aging. Total number per ROI of p16^+^, p21^+^, and p53^+^ neurons (A–F), microglia (G–L), and astrocytes (M–R) in hippocampus and cortex at 4, 12, and 24 months in WT and *App* ^*NL‐G‐F*^ mice. Significant age‐ and genotype‐related differences were detected in microglia and astrocytes, while neuronal expression remained largely unchanged. Data are expressed as total cell counts normalized per tissue area to assess the absolute senescent cell burden. All quantifications were performed on the same tissue sections and staining procedures reported in Figures 1, 2, 3. Data are shown as mean ± SEM. Statistical analysis: Two‐way ANOVA. *p* < 0.05 **p < 0.01 ***p < 0.001 WT4/12/24 versus AppNLGF4/12/24; ^ *p* < 0.05 ^^ *p* < 0.01 ^^^*p* < 0.001 AppNLGF4 versus AppNLGF12/24; # *p* < 0.05 # # *p* < 0.01 # # # *p* < 0.001 WT4 versus WT12/24.

This analysis shows that the neuronal expression levels of p16, p21, and p53 are unaffected by genotype, as there were no significant differences between WT and *App* ^*NL‐G‐F*^ mice (Figure 4A–F). In contrast, both p16 and p21 exhibited age‐related changes in neurons. The number of p16^+^ neurons in the hippocampus significantly depended on age (two‐way ANOVA: *F*(2,19) = 39.14, *p* = 0.0001), with no significant effect of genotype (*F*(1,19) = 0.022, *p* = 0.884) and no interaction (*F*(2,19) = 0.229, *p* = 0.798). Post hoc tests (Tukey's test) found that p16+ neuron numbers were significantly lower at 12 months than at 4 months, in both WT (*p* = 0.0002) and *App* ^*NL‐G‐F*^ mice (*p* = 0.0002) (Figure 4A). In the cortex, a similar significant age effect was observed (*F*(2,19) = 44.65, *p* = 0.0001), with no effect of genotype (*F*(1,19) = 0.378, *p* = 0.546) and no interaction (*F*(2,19) = 1.203, *p* = 0.322). At 12 months, WT mice had significantly fewer p16^+^ neurons than at 4 months (*p* = 0.0046), a pattern similar to that observed in the hippocampus (Figure 4B). Regarding p21^+^ neurons in the hippocampus, a significant main effect of age was found (two‐way ANOVA: *F*(2,19) = 16.08, *p* = 0.0001), with no significant effect of genotype (*F*(1, 19) = 1.108, *p* = 0.306) and no interaction (*F*(2,19) = 2.547, *p* = 0.105). Post hoc analysis showed that p21+ neuron counts were significantly higher at 24 months than at 4 months in *App* ^*NL‐G‐F*^ mice (*p* = 0.0012; Figure 4C). In the cortex, a similar significant age effect was observed (two‐way ANOVA: *F*(2,19) = 29.15, *p* = 0.0001; genotype: *F*(1,19) = 0.493, *p* = 0.491; interaction: *F*(2,19) = 0.575, *p* = 0.572), with both WT (*p* = 0.0004) and *App* ^*NL‐G‐F*^ (*p* = 0.0007) mice showing higher p21^+^ neuron counts at 24 months compared to 4 months (Figure 4D). The number of p53^+^ neurons did not show significant age‐related differences in either genotype (hippocampus: *F*(2,18) = 7.019, *p* = 0.006 for age, but no significant post hoc pairwise comparisons; cortex: *F*(2,18) = 1.965, *p* = 0.169; Figure 4E,F).

In contrast, the number of microglia expressing p16 and p21 was significantly higher in *App* ^*NL‐ G‐ F*^ mice compared to WT. For p16^+^ microglia in the hippocampus, two‐way ANOVA showed a significant genotype effect (*F*(1,19) = 31.66, *p* = 0.0001), an age effect (*F*(2,19) = 19.08, *p* = 0.0001), and a significant interaction (*F*(2,19) = 11.17, *p* = 0.0006). Post‐hoc tests confirmed that *App* ^*NL‐G‐F*^ mice had more p16^+^ microglia than WT at both 12 months (*p* = 0.041) and 24 months (*p* = 0.0001; Figure 4G). A similar pattern appeared in the cortex, where genotype (*F*(1,19) = 30.04, *p* = 0.0001), age (*F*(2,19) = 11.56, *p* = 0.0005), and their interaction (*F*(2,19) = 8.341, *p* = 0.0025) all showed significance, with *App* ^*NL‐G‐F*^ mice having higher p16^+^ microglial counts at 12 (*p* = 0.022) and 24 months (*p* = 0.0001) compared to WT (Figure 4H). For p21^+^ microglia in the hippocampus, two‐way ANOVA indicated significant effects of genotype (*F*(1,19) = 30.04, *p* = 0.0001), age (*F*(2,19) = 15.69, *p* = 0.0003), and their interaction (*F*(2,14) = 9.021, *p* = 0.003). Post hoc comparisons revealed more p21^+^ microglia in *App* ^*NL‐G‐*F^ than WT at 12 months (*p* = 0.005) and 24 months (*p* = 0.0001; Figure 4I). In WT mice, aging effects were significant only in the hippocampus at 24 months, with increased p21^+^ microglia compared to 4 months (*p* = 0.036; Figure 4I). In the cortex, effects of genotype (*F*(1,15) = 24.08, *p* = 0.0002), age (*F*(2,15) = 10.57, *p* = 0.0014), and their interaction (*F*(2,15) = 4.751, *p* = 0.025) were observed, with *App* ^*NL‐G‐ F*^ mice showing higher p21^+^ microglial counts at 12 (*p* = 0.018) and 24 months (*p* = 0.001) compared to WT (Figure 4J). The number of p53^+^ microglia in the hippocampus was also significantly increased in *App* ^*NL‐G‐F*^ mice, with two‐way ANOVA showing effects of genotype (*F*(1,19) = 359.2, *p* = 0.0001), age (*F*(2,19) = 434.1, *p* = 0.0001), and a highly significant interaction (*F*(2,19) = 240.7, *p* = 0.0001). Post hoc tests confirmed that *App* ^*NL‐G‐F*^ mice had more p53^+^ microglia than WT at 12 months (*p* = 0.0069) and 24 months (*p* = 0.0001; Figure 4K). In the cortex, effects of genotype (*F*(1,19) = 74.68, *p* = 0.0001), age (*F*(2,19) = 90.00, *p* = 0.0001), and their interaction (*F*(2,19) = 59.59, *p* = 0.0001) were significant. Post‐hoc comparison revealed differences only at 24 months (*p* = 0.0001; Figure 4L).

In WT mice, the number of p16^+^ astrocytes in the hippocampus decreases significantly with age, as shown by two‐way ANOVA (age effect: *F*(2,17) = 26.13, *p* = 0.0001; genotype: *F*(1,17) = 0.006, *p* = 0.940; interaction: *F*(2,17) = 0.600, *p* = 0.560). Post hoc tests indicated that WT mice had significantly fewer p16+ astrocytes at 12 months (*p* = 0.0006) and 24 months (*p* = 0.006) than at 4 months. A similar decline was observed in *App* ^*NL‐G‐F*^ mice at 12 months compared with 4 months (*p* = 0.0011; Figure 4M). No age‐related changes in p16 expression appeared in cortical astrocytes (age effect: *F*(2,19) = 4.381, *p* = 0.027; interaction: *F*(2,19) = 0.646, *p* = 0.535; Figure 4N). A comparable trend was observed for p21 in hippocampal astrocytes of WT mice. Two‐way ANOVA showed a significant interaction between age and genotype (*F*(2,19) = 9.512, *p* = 0.0014), a significant age effect (*F*(2,19) = 6.353, *p* = 0.008), and a significant genotype effect (*F*(1,19) = 12.22, *p* = 0.002). Post hoc analysis revealed that the total number of p21^+^ astrocytes was significantly lower at 12 months than at 4 months in WT mice (*p* = 0.009; Figure 4O). Additionally, at 12 months of age, *App* ^*NL‐G‐F*^ mice had a significantly higher number of p21^+^ astrocytes compared to WT mice of the same age (*p* = 0.0003), although this trend did not persist at 24 months (Figure 4O). In the cortex, two‐way ANOVA revealed significant effects of age (*F*(2,19) = 48.74, *p* = 0.0001), genotype (*F*(1,19) = 71.37, *p* = 0.0001), and their interaction (*F*(2,19) = 8.261, *p* = 0.003). Post hoc tests confirmed an increase in p21+ astrocytes with age in WT mice at 24 months (*p* = 0.004) and in *App* ^*NL‐G‐F*^ mice at both 12 (*p* = 0.002) and 24 months (*p* = 0.0001). Moreover, there was a significant Alzheimer's disease (AD)‐related increase at 12 (*p* = 0.0001) and 24 months (*p* = 0.0001; Figure 4P). p53^+^ astrocytes in the hippocampus increased significantly with age in both WT and *App* ^*NL‐G‐F*^ mice. Two‐way ANOVA indicated a significant age effect (*F*(2,18) = 37.77, *p* = 0.0001), but no significant effect of genotype (*F*(1,18) = 0.130, *p* = 0.722) or interaction (*F*(2,18) = 1.044, *p* = 0.373). Post hoc analysis confirmed significant increases at 24 months versus 4 months in both WT (*p* = 0.033) and *App* ^*NL‐G‐F*^ (*p* = 0.042) mice (Figure 4Q). In the cortex, two‐way ANOVA found significant effects of age (*F*(2,18) = 24.34, *p* = 0.0001) and genotype (*F*(1,18) = 10.03, *p* = 0.005), with a non‐significant interaction (*F*(2,18) = 2.693, *p* = 0.095). Post hoc tests showed a significant difference between genotypes at 24 months (*p* = 0.021), and an increase in *App* ^*NL‐G‐F*^ mice at 24 months compared to 4 months (*p* = 0.0004) and 12 months (*p* = 0.0006; Figure 4R).

### 3.5 Gene Expression and Protein Evidence of p16‐Driven Senescence, IL‐1β Upregulation, and Lysosomal Dysfunction in *App* ^*NL‐G‐F*^ Mouse Brain

**FIGURE 5:**
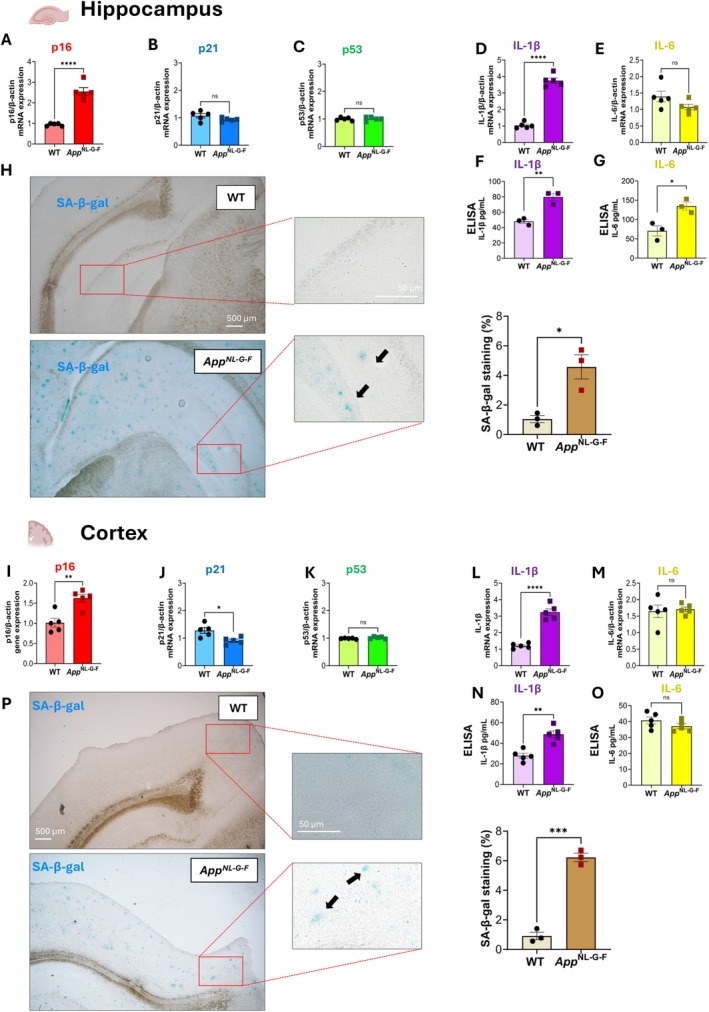
qPCR, ELISA analysis of senescence and inflammatory markers, and SA‐β‐Galactosidase staining at 12 months of age. Relative mRNA expression of p16 (A, I), p21 (B, J), and p53 (C, K) in hippocampus and cortex of WT (*n* = 5) and *App* ^*NL‐G‐F*^ (*n* = 5) mice. mRNA levels of IL‐1β (D, L) and IL‐6 (E, M) in hippocampus (*n* = 5 for group) and cortex (*n* = 5 for group), and ELISA quantification of IL‐1β (F, N) and IL‐6 (G, O) in hippocampus (*n* = 3 for group) and cortex (*n* = 5 for group) of WT and *App* ^*NL‐G‐F*^ mice. p16 mRNA, IL‐1β mRNA and protein levels were significantly increased in *App* ^*NL‐G‐F*^ mice. Representative images and quantification of SA‐β‐gal activity showing increased senescence‐associated β‐galactosidase staining in hippocampus (H) and cortex (P) of *App* ^*NL‐G‐F*^ (*n* = 3) mice compared to WT (*n* = 3) mice. Scale bars: 500 μm and 50 μm. Statistical analysis: Unpaired *t*‐test. *p < 0.05. All data are presented as mean ± SEM. *p < 0.05, **p < 0.01, ***p < 0.001; ****p < 0.0001 versus WT group.

Furthermore, gene expression levels of p16, p21, and p53 were assessed using qPCR analysis of the mouse brain at 12 months old. A significant increase in p16 mRNA was found in both the hippocampus (*F*(4,4) = 32.14, *p* = 0.0001) and cortex (*F*(4,4) = 1.29, *p* = 0.003) of *App* ^*NL‐G‐F*^ mice compared to WT controls (Figure 5A,I). In contrast, p21 gene expression in the hippocampus was similar between the groups (*F*(4,4) = 7.44, *p* = 0.134) (Figure 5B), but a significant decrease was observed in the cortex of *App* ^*NL‐G‐F*^ mice (*F*(4,4) = 2.98, *p* = 0.0001) (Figure 5J). No differences in p53 gene expression were observed between groups in both the hippocampus (*F*(4,4) = 1.41, *p* = 0.486) and cortex (*F*(4,4) = 3.55, *p* = 172) (Figure 5C,K).

Additionally, to assess the presence of SASP, the gene expression and protein levels of the pro‐inflammatory cytokines IL‐1β and IL‐6 were analyzed (Figure 5D–G,L–O). IL‐1β gene expression was significantly increased in the hippocampus of *App* ^*NL‐G‐F*^ mice (*F*(4,4) = 3.93, *p* = 0.0001) (Figure 5D) and in the cortex (*F*(4,4) = 8.68, *p* = 0.0001) (Figure 5L) compared to WT controls, as shown by qPCR analyses. Conversely, IL‐6 gene expression levels were similar between WT and *App* ^*NL‐G‐F*^ mice in both the hippocampus (*F*(4,4) = 3.72, *p* = 0.116) (Figure 5E) and cortex (*F*(4,4) = 7.1, *p* = 0.777) (Figure 5M). Consistent with the gene expression data, ELISA analysis revealed higher IL‐1β protein levels in the *App* ^*NL‐G‐F*^ mouse cortex (*F*(4,4) = 1.88, *p* = 0.001) (Figure 5N) and hippocampus (*F*(2,2) = 3.42, *p* = 0.004) (Figure 5F) compared to WT controls, whereas IL‐6 levels were increased only in the hippocampus (*F*(2,2) = 1.63, *p* = 0.0017) (Figure 5G), with no differences observed in the *App* ^*NL‐G‐F*^ mouse cortex compared to WT controls (*F*(4,4) = 2.00, *p* = 0.215) (Figure 5O).

To assess lysosomal dysfunction, senescence‐associated β‐galactosidase (SA‐β‐GAL) activity was measured. A significant increase in SA‐β‐GAL activity was observed in the hippocampus (*F*(2,2) = 11.10, *p* = 0.014) (Figure 5H) and cortex (*F*(2,2) = 1.22, *p* = 0.0002) (Figure 5P) of *App* ^*NL‐G‐F*^ mice, further indicating the presence of senescent cells in these mice at 12 months old. Additionally, double staining of SA‐β‐gal+Iba1 revealed partial colocalization of SA‐β‐gal and microglia cells (Figure S1B).

### 3.6 Morphology and Plaque‐Association of Senescent Microglia

The previous data indicate that microglia are one of the most significantly affected senescent cell types in *App* ^*NL‐G‐F*^ mice, along with an increase in their proliferation (Figure 2A,B). Morphological alterations are also considered characteristic of senescent cells (Storer et al. 2013). To investigate morphological differences in microglia, we analyzed microglial ramification using skeletal analysis and assessed microglial complexity, shape, and size through fractal analysis in WT and *App* ^*NL‐G‐F*^ mice at 12 months of age. Skeletal analysis, performed on Iba1^+^ cells, revealed significant differences between the two genotypes. WT mice exhibited a higher number of endpoints per cell and longer process lengths per cell compared to *App* ^*NL‐G‐F*^ mice in both the hippocampus (*F*_endpoint_s(4,4) = 83.35, *p* = 0.016; *F*_process_(4,4) = 15.69, *p* = 0.020) and cortex (*F*_endpoint_s(4,4) = 20.96, *p* = 0.016; *F*_process_(4,4) = 30.91, *p* = 0.028) (Figure 2C,D).

**FIGURE 6:**
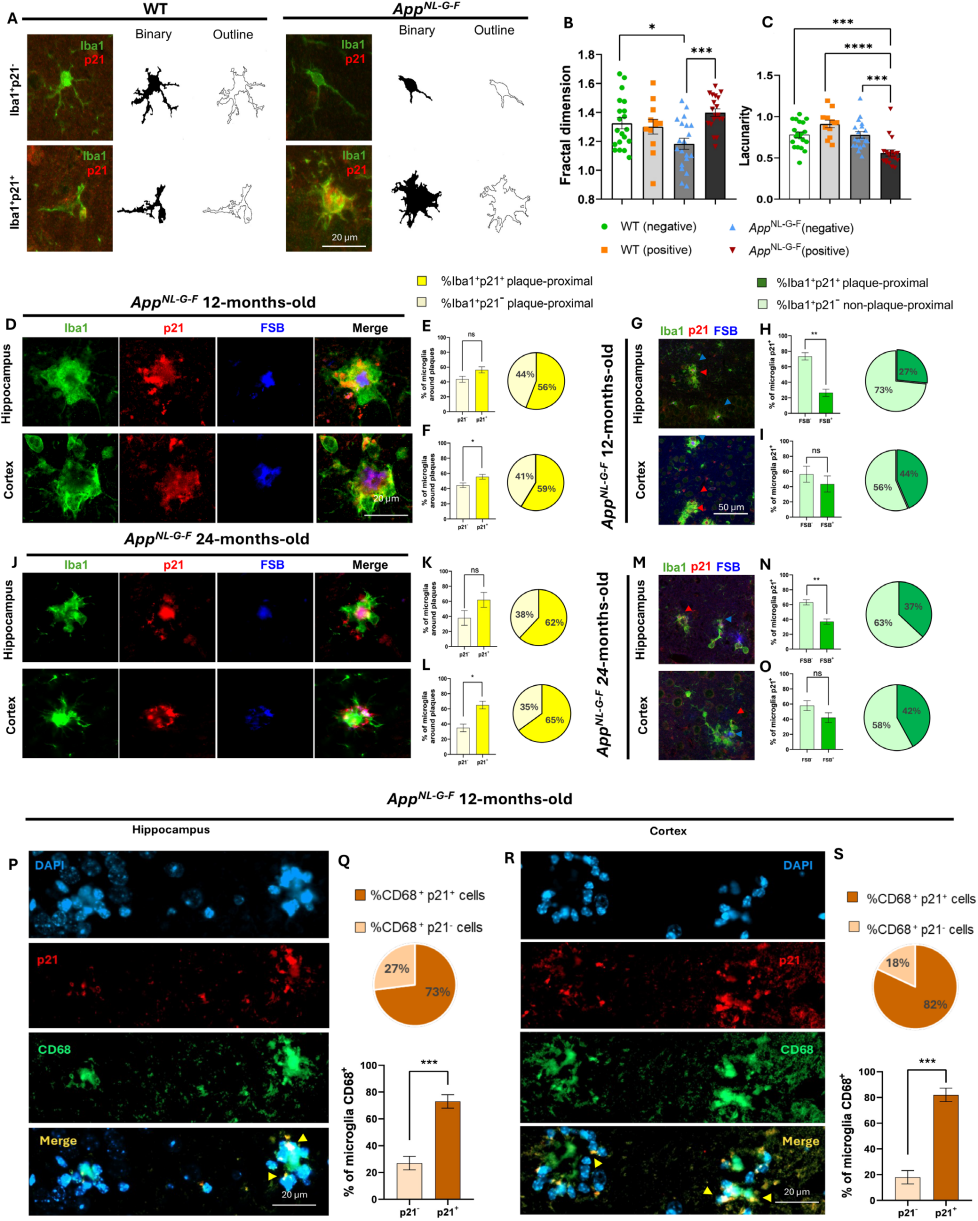
Microglial morphological analysis, association with amyloid plaques at 12 and 24 months of age, and phenotype characterization. Representative images of microglial Fractal analysis in hippocampus of WT (*n* = 4; total 20 cells) and *App* ^*NL‐G‐F*^ (*n* = 4; total 20 cells) mice (A). Microglia p21^+^ and p21^−^ from the two genotypes were considered. Fractal dimension was found to be significantly increased in p21^+^ microglia of *App* ^*NL‐G‐F*^ mice compared to p21^−^ microglia of *App* ^*NL‐G‐F*^ (B); Lacunarity showed a significant decrease in p21^+^ microglia of *App* ^*NL‐G‐F*^ compared to all the other groups considered (C). Microglial association with amyloid plaques in hippocampus and cortex of 12 (D, G) and 24‐month‐old (J, M) mice. At 12 months of age, data showed that 56% of the microglia surrounding plaques in the hippocampus (E) and 59% in the cortex (F) were p21^+^. The plaque‐associated p21^+^ microglia represented 27% of the total p21^+^ microglial population in the hippocampus (H) and 44% in the cortex (I). In 24‐month‐old mice, plaque‐associated p21^+^ represented 62% (K) in hippocampus and 65% (L) in cortex, corresponding to 37% (N) in hippocampus and 42% (O) in cortex of the total microglia population. Characterization of senescent microglia phenotype in hippocampus (P) and cortex (R). Data showed that 73% of CD68^+^ microglia were also p21^+^ in hippocampus (Q), accounting also for the 82% in the cortex (S). Scale bar: 20 μm. Data are shown as mean ± SEM. Statistical analysis: One‐way ANOVA. *p < 0.05, ***p < 0.001; ****p < 0.0001.

Fractal analysis (Figure 6A) showed a significant effect of group on fractal dimension (one‐way ANOVA: *F*(3,68) = 6.259, *p* = 0.0008). Post hoc Tukey tests revealed that fractal dimension was significantly lower in p21^−^ microglia from *App* ^*NL‐G‐F*^ mice compared to p21^−^ microglia from WT mice (*p* = 0.032) (Figure 6B). Additionally, p21^+^ microglia from *App* ^*NL‐G‐F*^ mice exhibited a significantly higher fractal dimension than p21^−^ microglia from the same genotype (*p* = 0.001), indicating notable morphological changes in senescent microglia (Figure 6B). Lacunarity also varied significantly among groups (one‐way ANOVA: *F*(3,66) = 12.827, *p* = 0.0001). Post hoc Tukey analysis showed that lacunarity was significantly reduced in p21^+^ microglia of *App* ^*NL‐G‐F*^ mice compared to p21^−^ microglia of the same genotype (*p* = 0.001), p21^+^ microglia of WT mice (*p* = 0.0001), and p21^−^ microglia of WT mice (*p* = 0.001) (Figure 6C).

To investigate the relationship between microglial senescence and the progression of amyloid pathology, we measured the percentage of p21+ microglia in the vicinity of Aβ plaques at 12 and 24 months of age, relative to the total number of microglia in the plaque vicinity. At 12 months, p21^+^ microglia represented 56% of plaque‐associated microglia in the hippocampus (*F*(3,3) = 1.00, *p* = 0.068) and 59% in the cortex. Notably, senescent microglia were the predominant population in the cortex at this age (*F*(3,3) = 1.00, *p* = 0.041) (Figure 6D–F). These plaque‐associated p21^+^ microglia represented only 27% of the total p21^+^ microglial population in the hippocampus, indicating that most senescent microglia are located outside the plaque vicinity (*F*(2,2) = 1.00, *p* = 0.002) (Figure 6G,H). In the cortex, this proportion was 44%, suggesting an almost equal distribution between p21^+^ plaque‐associated and non‐associated microglia (*F*(2,2) = 1.00, *p* = 0.438) (Figure 6G–I). At 24 months, in the hippocampus p21^+^ microglia comprised 62% of the plaque‐associated microglial population (*F*(3,3) = 1.00; *p* = 0.133), while in the cortex, they made up 65% (*F*(3,3) = 1.00, *p* = 0.005) (Figure 6J–L). These accounted for 37% of the total p21^+^ microglial population in the hippocampus (*F*(3,3) = 1.00; *p* = 0.005), and 42% in the cortex, indicating an almost equal distribution inside and outside the plaque vicinity (*F*(3,3) = 1.00; *p* = 0.129) (Figure 6M–O). Additionally, we analyzed p16 as a senescence marker. In 12‐month‐old mice, 28% of plaque‐associated microglia in the hippocampus and 43% in the cortex were p16^+^ (Figure 3A,C,E), corresponding to 16% and 24% of the total p16^+^ microglial populations in each region, respectively (Figure 3A,B,D). Similar data persisted at 24 months, with 28% of microglia around plaques in the hippocampus and 46% in the cortex of 24‐month‐old mice were also plaque‐associated (Figure 3F,H,J). Specifically, the total microglia p16^+^, accounted for 17% in the hippocampus and 21% in the cortex (Figure 3F,G,I).

Additionally, to gain a deeper understanding of the senescent microglia phenotype, we examined the co‐expression of p21 and CD68 in 12‐month‐old *App* ^*NL‐G‐F*^ mice. Our analysis revealed that most CD68^+^ microglia also express p21, with 73% in the hippocampus (*F*(3,3) = 1, *p* = 0.0007) (Figure 6P,Q), and 82% in the cortex (*F*(3,3) = 1, *p* = 0.0001) (Figure 6R,S). This indicates that a significant portion of reactive microglia in these regions exhibit a senescent phenotype.

## 4 Discussion

The findings of this study reveal that cellular senescence markers increase with age in the *App* ^*NL‐G‐F*^ mouse model of AD. The differences in the expression levels of p16, p21, and p53 across neurons, microglia, and astrocytes suggest that aging may have distinct effects on each cell type and may contribute distinctly to the progression of AD. Neuronal senescence has been associated with impaired synaptic function and altered gene expression (Mertens et al. 2021), while senescent microglia and astrocytes are known to promote chronic neuroinflammation by releasing pro‐inflammatory cytokines and SASP factors (Sikora et al. 2021). Aging, the primary risk factor for AD, is intrinsically linked to the accumulation of senescent cells throughout the body, including the brain (Kritsilis et al. 2018). Previous studies have identified senescent cells in the brains of both AD patients and animal models, highlighting the impact of this cellular state on neurodegeneration (Li et al. 2022; Zhu et al. 2024). The progressive increase in senescence markers observed here supports a role for senescence in the disease process, potentially contributing to the gradual cognitive decline characteristic of AD and extensively observed in *App* ^*NL‐G‐F*^ mice (Locci et al. 2021; Mehla et al. 2019). Together, these findings support the hypothesis that amyloid pathology can directly induce cellular senescence in multiple brain cell types, reinforcing the translational relevance of the *App* ^*NL‐G‐F*^ model for studying senescence in AD.

Microglia and astrocytes emerged as the main senescent cell types in this amyloid‐driven mouse model, exhibiting pronounced increases in p16 and p21 co‐expression, elevated SA‐β‐gal activity, increased IL‐1β and IL‐6 levels, and distinct morphological changes. In contrast, no genotype‐dependent differences in neuronal senescence markers were observed at any examined age, even after 24 months in *App* ^*NL‐G‐F*^ mice. The induction of neuronal senescence might require additional pathological factors, such as intracellular tau aggregation, aligning with studies that connect neuronal senescence to tau pathology in human AD brains and tau transgenic models (Bussian et al. 2018; Dehkordi et al. 2021; Musi et al. 2018). Although our findings do not exclude other neuron‐related senescence events, further research is needed to clarify potential indirect mechanisms linking Aβ pathology to neuronal senescence.

The increased presence of senescent microglia indicates a strong connection between the progression of amyloid pathology and microglial aging. However, the data does not clarify whether microglial senescence results from AD pathology or actively contributes to its development. Senescent microglia are generally understood to drive a harmful cycle of chronic neuroinflammation, showing impaired phagocytic ability, which limits their capacity to clear amyloid‐beta plaques, tau aggregates, and cellular debris—processes vital for maintaining brain health (Ritzel et al. 2015). Additionally, senescent microglia tend to release higher levels of pro‐inflammatory cytokines and other factors that make up the SASP (Sikora et al. 2021). This increased pro‐inflammatory environment can worsen neuroinflammation, a key feature of AD, potentially leading to neuronal dysfunction and loss. IL‐1β and IL‐6 are two well‐known interleukins linked to SASP. In this study, higher levels of IL‐1β in both the cortex and hippocampus and elevated IL‐6 in the hippocampus support the idea that SASP‐associated interleukins are upregulated in senescence and may contribute to neuroinflammation.

The significant upregulation of p21 observed in the *App* ^*NL‐G‐F*^ microglia is particularly noteworthy, as p21 is a strong marker of microglial senescence. Senescent p21^+^ microglia in *App* ^*NL‐G‐F*^ mice have been found across the tissue areas, not solely localized around amyloid plaques, suggesting that the presence of these senescent immune cells in the *App* ^*NL‐G‐F*^ mouse model is not exclusively dependent on direct contact with amyloid plaque deposits. Nevertheless, the higher proportion of plaque‐associated p21^+^ microglia in the cortex compared to the hippocampus (44% vs. 27%) aligns with the earlier documented amyloid burden in the cortex (Saito et al. 2014), indicating region‐specific patterns of microglial senescence driven by local amyloid burden, an aspect that remains largely unexplored. We hypothesize that soluble amyloid‐beta, rather than plaques themselves, may be the primary driver of senescence in microglia. Interactions involving soluble amyloid‐beta could be key to the accumulation of senescent microglia in the *App* ^*NL‐G‐F*^ model. This has been demonstrated in vitro, where Aβ, in a dose‐dependent manner, induced microglial senescence, notably upregulating p21 (An et al. 2022). Although overall senescence marker levels were low at 4 months, a significant increase in p21^+^ microglial cells was observed in the cortex, indicating an early onset of microglial senescence associated with developing amyloid pathology. Previous studies in the less aggressive *App* ^*NL‐F*^ model have reported early changes in senescence markers at the mRNA level (Fang et al. 2024; McFadden et al. 2026). However, direct comparisons are limited by differences in methods (gene expression vs. immunohistochemistry) and by genetic differences between the A*pp* ^*NL‐F*^ and *App* ^*NL‐G‐F*^ lines in the Aβ species produced and their aggregation kinetics.

Morphological analyses further support a senescent microglial phenotype in *App* ^*NL‐G‐F*^ mice. The less ramified morphology observed, evidenced by decreased endpoints per cell and shorter process length, along with genotype‐specific alterations in fractal dimension and lacunarity within the p21^+^ population, provide strong evidence for altered microglial function in amyloid pathology. These structural changes contribute to the broader picture of microglial dysregulation in AD, raising important questions about how this senescent phenotype relates to other recognized microglial states, such as Disease‐Associated Microglia (DAM). Although the distinction between DAM and senescent microglia remains unclear, previous studies using the 5xFAD mouse model have shown that senescent microglia have a unique protein profile that differs from DAM, including classical senescence markers and homeostatic microglial proteins (Rachmian et al. 2024). Similarly, transcriptomic analyses in P301S (PS19) tauopathy mice indicated that only a subset of DAM displays features of senescence, with the overlap mainly driven by shared inflammatory signatures (Ng, Zhang, et al. 2023). These findings highlight the complexity and diversity of microglial responses in AD models. It is possible that senescent microglia develop as a downstream state of DAM within a common pathway, or they may arise independently as a separate response to stress. Notably, most microglia positive for CD68, a lysosomal marker of microglial reactivity, co‐expressed p21 in both the hippocampus (73%) and cortex (82%), indicating that reactive microglia in *App* ^*NL‐G‐F*^ mice mainly show a senescent phenotype, further supporting the idea that microglial senescence and neuroinflammatory activation are closely linked processes in the context of amyloid pathology.

Comparison across different AD mouse models with varied pathological profiles supports a mechanism where senescence is induced specifically by pathology. The *App* ^*NL‐G‐F*^ and 5xFAD models, both characterized by mainly amyloid pathology, mostly show glial senescence (Rachmian et al. 2024), while the PS19 tauopathy model exhibits neuronal senescence marked by p16/p21 expression and cellular dysfunction (Bussian et al. 2018; Graves et al. 2025). The differing patterns of senescence in these models support a mechanism where amyloid pathology mainly causes glial senescence, as seen in *App* ^*NL‐G‐F*^ and 5xFAD, whereas tau pathology seems necessary for strong neuronal senescence, as observed in PS19 and human AD with neurofibrillary tangles. This distinction has important implications for therapy, indicating that senotherapeutic strategies may need to be tailored based on the dominant pathological protein and the affected cell type at various stages of the disease.

The astrocyte senescence signature observed in this study was characterized by p21 and p53 upregulation without a corresponding increase in p16. Notably, p21^+^ astrocytes showed a particularly strong genotype‐dependent difference at 12 months. This profile contrasts with in vitro findings, where Aβ42‐exposed astrocytes exhibited p16 upregulation and increased SA‐β‐gal activity (Bhat et al. 2012), suggesting that progressive amyloid pathology may activate different senescence pathways compared to acute Aβ42 exposure in isolated cell cultures. Elevated astrocyte senescence has also been seen in human aging and AD brains (Meldolesi 2023), supporting the relevance of these in vivo findings.

Beyond the specific cell‐type changes seen in *App* ^*NL‐G‐F*^ mice, the significant increase of SA‐β‐GAL activity in both hippocampal and cortical areas offers supporting evidence for widespread cellular senescence in this mouse model. This result is especially important because SA‐β‐GAL is a well‐known biomarker of lysosomal enlargement and dysfunction, which are characteristic features of senescent cells involved in their pro‐inflammatory and proteolytic imbalance (Lee et al. 2006).

Overall, this study provides valuable insights into the distinct patterns of expression for key senescence markers p16, p21, and p53 across different ages and cell types in the *App* ^*NL‐G‐F*^ mouse model. The significant increase of p16 and p21 levels at both 12 and 24 months of age, especially within glial cells, emphasizes the importance of these two markers in the context of AD‐related senescence, suggesting that the observed senescence is likely mediated through pathways involving these key cell cycle regulators. In contrast, immunofluorescence data for p53, a tumor suppressor protein known for its role in DNA damage response, cell cycle arrest, apoptosis, and senescence, showed cell‐type‐specific increases in the marker, particularly in hippocampal astrocytes at 12 months and in microglia at 24 months of age. These results highlight the importance of examining senescence markers at the single‐cell and subcellular levels. They suggest that although p53 might not be globally upregulated in the brain of the *App* ^*NL‐G‐F*^ mice, it could still play a significant role in inducing or maintaining senescence in specific cell populations influenced by AD pathology.

The pattern of concurrent p16 and p21 upregulation with mostly unchanged p53 levels aligns with a model of chronic stress‐induced senescence rather than acute DNA damage (Ng, McNeely, and Baker 2023), consistent with the persistent oxidative and inflammatory environment typical of amyloid pathology (Walton et al. 2020). Likely upstream regulators include the TGF‐β/SMAD and NF‐κB signaling pathways, both known to promote p16 and p21 transcription and reinforce the SASP (Chien et al. 2011; Ueda et al. 2021). Elevated IL‐1β and IL‐6 levels in our study further support NF‐κB–mediated SASP activation. Additionally, sustained oxidative and lysosomal stress might activate the ATM–Chk2 and mTOR–TFEB pathways, resulting in mitochondrial dysfunction, lysosomal enlargement, and increased SA‐β‐GAL activity (Ma et al. 2018). The lack of p53 increase at early time points suggests that microglial senescence begins through p53‐independent mechanisms, possibly involving alternative stress‐activated pathways like p38‐MAPK signaling (Freund et al. 2011; Herranz and Gil 2018), with p21 induction probably driven by upstream regulators other than p53 (Ćmielová and Řezáčová 2011). The late‐stage p53 accumulation observed at 24 months may be a secondary response to genotoxic damage accumulation in chronically senescent cells rather than a primary trigger of senescence (Rufini et al. 2013).

These findings align with human postmortem AD data, where single‐nucleus transcriptomics has identified premature senescence mainly in glial cells with limited evidence for neuronal senescence (Fancy et al. 2024), and with the recent observation that senescent border‐associated macrophages can spread senescence to nearby microglia via migrasomes during aging (Hu et al. 2025), further emphasizing the central role of microglial senescence in AD progression.

Some limitations of this study should be acknowledged; all experiments were conducted in female mice only, which limits conclusions about sex‐dependent differences in senescence responses. Although female *App* ^*NL‐G‐F*^ mice develop amyloid pathology similar to males (Benitez et al. 2021, et al. 2020; Daniels et al. 2023), female sex has been linked to increased susceptibility to DNA damage and a higher likelihood of senescence onset (Ng and Hazrati 2022), raising the possibility that the glial‐predominant senescence profile observed here may not fully apply to male groups. Future research including male mice is therefore needed. Additionally, senescence was measured using established immunohistochemical markers (p16, p21, p53, and SA‐β‐GAL), which only capture certain aspects of the senescent state and do not represent its full functional diversity; complementary transcriptomic profiling or functional SASP assays would offer deeper mechanistic insights. Lastly, whether glial senescence causes or results from amyloid pathology remains to be determined, and future studies using genetic, pharmacological, and cell‐specific approaches will be essential for clarifying its causal role in neurodegeneration.

These findings highlight the potential of *App* ^*NL‐G‐F*^ mice as a valuable platform for evaluating senescence‐targeted therapies in AD. Senolytic drugs have shown encouraging results in early‐phase clinical trials for other age‐related conditions, demonstrating their feasibility and potential to reduce the burden of senescent cells in vivo (Bussian et al. 2018; Hickson et al. 2019; Justice et al. 2019). Notably, a Phase I clinical trial investigating the use of the senolytic combination of Dasatinib and quercetin in patients with early‐stage symptomatic AD showed that these drugs can penetrate the central nervous system and have an acceptable safety profile (Gonzales et al. 2023). This provides critical proof‐of‐concept for the potential of senolytics in treating AD. While dasatinib and quercetin (D + Q) represent promising senolytic candidates for AD, recent studies have highlighted that their efficacy may vary considerably depending on sex and amyloid status. In particular, D + Q treatment had minimal effects in male C57BL/6 mice but was detrimental in females, leading to increased SASP expression, reduced energy metabolism, and impaired cognitive performance (Fang et al. 2023). Conversely, in the *App* ^*NL‐F*^ amyloidogenic model, D + Q treatment in female mice improved spatial memory, reduced amyloid burden, and decreased senescent cell markers (Fang et al. 2024). Beyond senolytics, senomorphic agents, which suppress SASP without eliminating senescent cells, represent a complementary strategy for reducing amyloid‐associated neuroinflammation. Given that microglia are identified here as the primary senescent population, therapeutic approaches specifically targeting senescent microglia, or combining senolytic and senomorphic strategies, may offer a more comprehensive approach to the complex pathology of AD.

Building on the findings of this study, several avenues for future research deserve attention. It is essential to investigate the specific upstream mechanisms that trigger cellular senescence in neurons, microglia, and astrocytes within the *App* ^*NL‐G‐F*^ model. This could involve examining the roles of Aβ oligomers, oxidative stress, DNA damage accumulation, and mitochondrial dysfunction. Evaluating the efficacy of various senolytic and senomorphic compounds in the *App* ^*NL‐G‐F*^ mouse model throughout different stages of disease progression would be a logical next step. Such studies could help identify the most promising therapeutic agents for targeting senescence in the context of AD pathology. Further research should focus on elucidating the functional consequences of senescence in specific brain cell types within this model. This could include assessing the impact of senescence on synaptic function, neuronal survival, and the ability of glial cells to clear amyloid‐beta and other pathological proteins.

## Author Contributions

Simone Tambaro, Eileen Mac Sweeney, Andrea Mastinu, Giulia Abate, and Daniela Uberti conceptualized and designed the experiments. Simone Tambaro and Eileen Mac Sweeney. wrote the initial draft of the manuscript. Andrea Mastinu, Giulia Abate, Daniela Uberti, and Per Nilsson critically revised and edited the manuscript. Eileen Mac Sweeney, Bjorn R.V. Bakker, and Simone Tambaro performed the experiments.

## Funding

This work was supported by Alzheimer's Association 24AARG‐1244398, Olle Engkvists Stiftelse 213‐0295, Gun & Bertil Stohnes Stiftelse, Demensfonden, Lindhés Advokatbyrå Stiftelse, Åhlén‐stiftelsen 223087, and Gamla tjänarinnor.

## Conflicts of Interest

The authors declare no conflicts of interest.

## Supporting information


**Figure S1:** Age‐Dependent Changes Grouped by Cell Type and Genotype, and SA‐βGal+Iba1 staining. (A) The percentage of positive cells has been averaged across all three Markers (p16, p21, and p53) and both Regions (Hippocampus and Cortex). This visualization clearly highlights the Genotype and Age effect on the three primary cell types. Microglia—and Astrocytes show a dramatic change in color from 4 to 12 months, confirming the accelerated pathology in these glial cell types that is effect of the genotype. The neurons WT row is showing a relatively high percentage of senescent markers already at 4 months and stays high in older ages, reinforcing the idea that baseline neural ‐marker expression is high regardless of the specific disease model, making the glial changes a much clearer finding. (B) Double staining of SA‐β‐gal+Iba1 showed a partial colocalization of SA‐β‐gal and microglia cells. Scale bars: 50 μm.
**Figure S2:** Microglial proliferation and Skeletal analysis of microglia in WT and *App* ^*NL‐G‐F*^ mice. Quantification of total microglia (Iba1^+^) in the hippocampus (A) and cortex (B) of WT and *App* ^*NL‐G‐F*^ mice at 4, 12, and 24 months of age. Data are expressed as total microglia per area. Data are shown as mean ± SEM. Statistical analysis: one‐way ANOVA. **p < 0.01; ****p < 0.0001 versus WT group. Quantification of the number of microglial endpoints per cell and process length per cell in the hippocampus and cortex of 12‐month‐old WT and *App* ^*NL‐G‐F*^ mice. Images were acquired at 20× magnification and analyzed using ImageJ software. Statistical analysis was performed using unpaired *t*‐tests. Data are presented as mean ± SEM. *p < 0.05 versus WT group.
**Figure S3:** Association of p16^+^ microglia with amyloid plaques at 12 and 24 months of age. The number of p16^+^ microglia and microglia surrounding amyloid plaques was assessed in both the hippocampus and cortex. At 12 months of age (A), data showed that 28% of the microglia surrounding plaques in the hippocampus (C) and 43% in the cortex (E) were p16^+^, corresponding to 16% (B) in hippocampus and 24% (D) in cortex of the total microglia population. In 24‐months‐old mice (F), data showed that 28% of the microglia surrounding plaques in the hippocampus (H) and 46% in the cortex (J) were p16^+^. The plaque‐associated p16^+^ microglia represented 17% of the total p16^+^ microglial population in the hippocampus (G) and 21% in the cortex (I). Data are expressed as total microglia per area. Scale bars: 20 μm. Data are presented as mean ± SEM. Statistical analysis: unpaired *t*‐test. ****p < 0.0001.

**Table S1:** List of the antibodies used in the study.

**Table S2:** Percentage of neurons, microglia and astrocytes positive for p16, p21 and p53 in WT and *App* ^*NL‐G‐F*^ mice at 4, 12 and 24 months of age. In the table are reported the percentage mean ± SEM.

## Data Availability

Data supporting this study are available on reasonable request to the corresponding authors.
