# Supplementary figures and images for: Cell Type‐Specific Expression of p16, p21, and p53 Reveals Age‐Dependent Glial Senescence in the AppNL‐G‐F Mouse Model of Alzheimer's Disease
Source: Aging Cell. 2026 Apr 14;25(4):e70478. doi: 10.1111/acel.70478 (PMC13078136; doi:10.1111/acel.70478)

Supplementary Figure 1

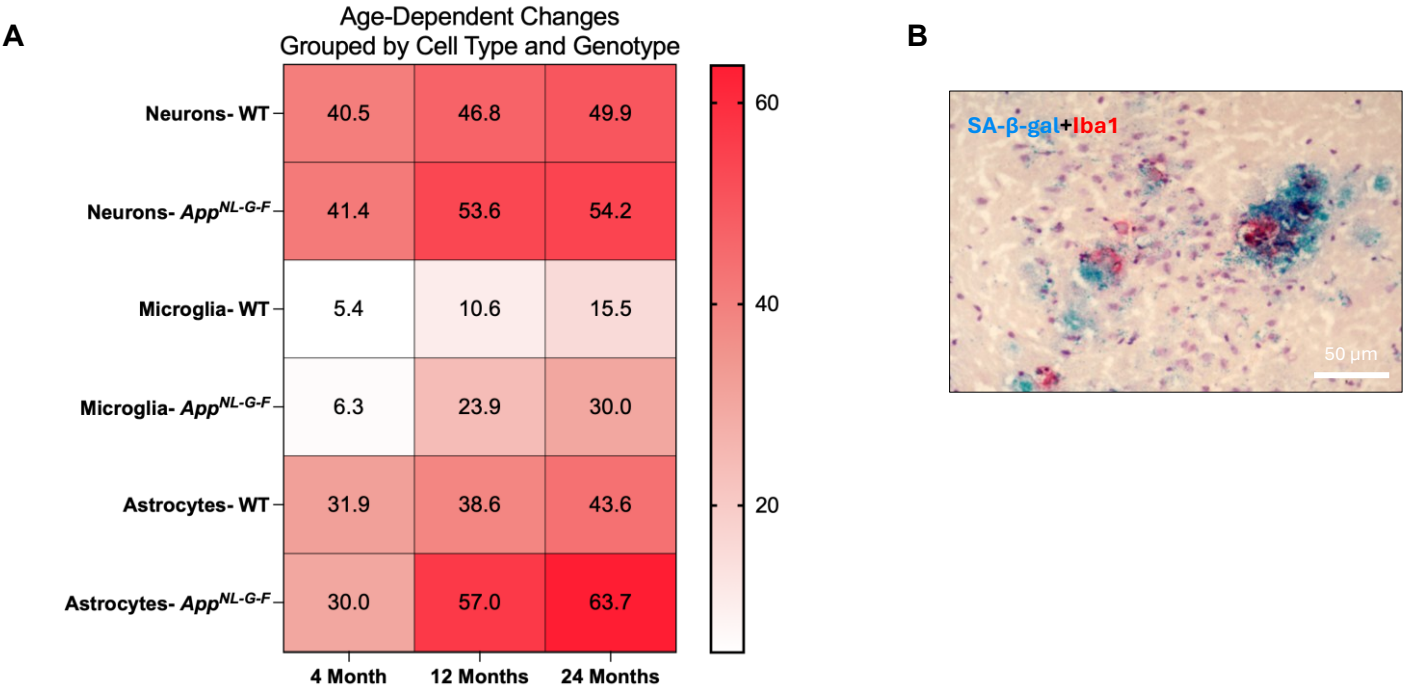

Supplementary Figure 2

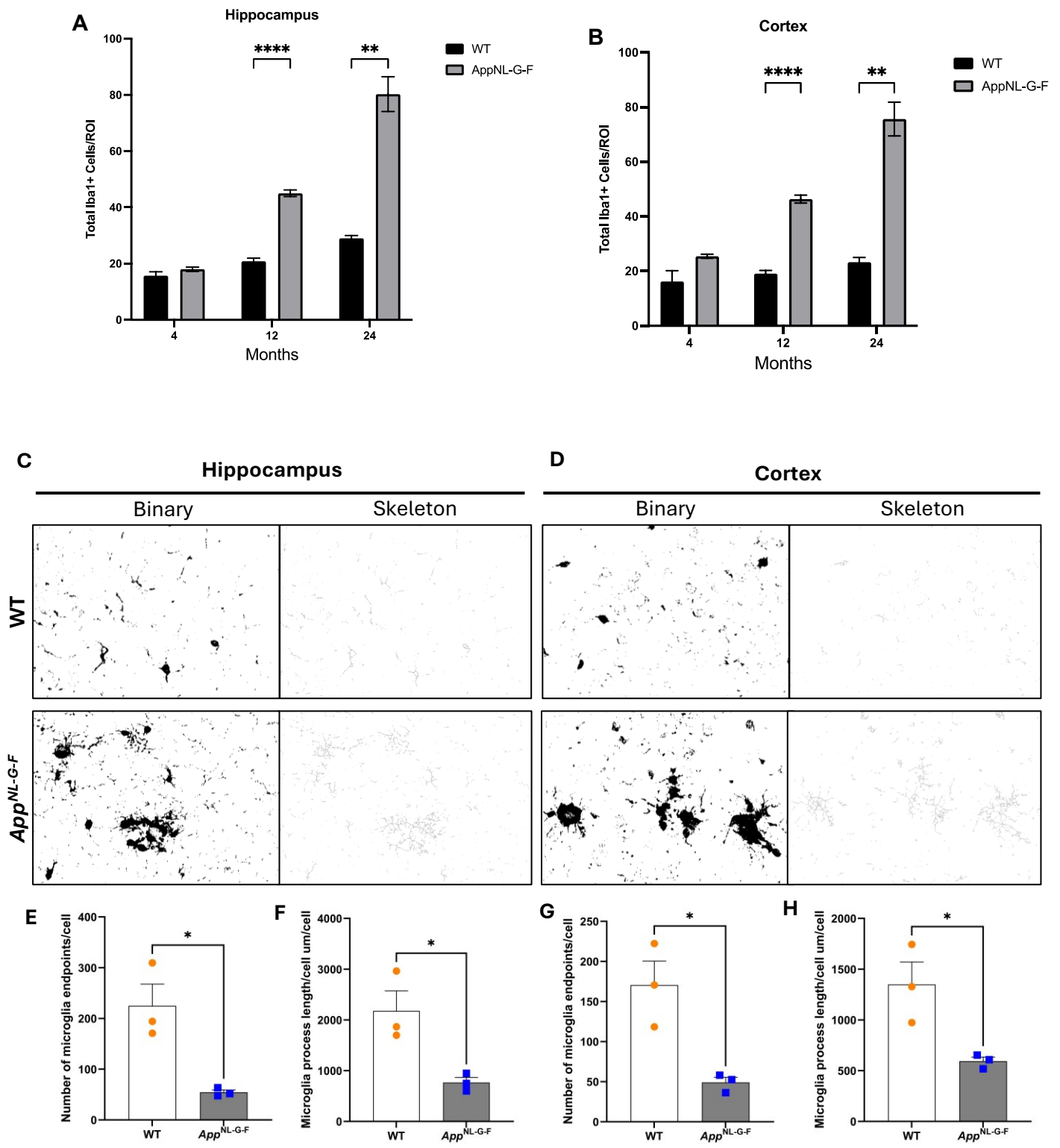

Supplementary Figure 3

12-months-old

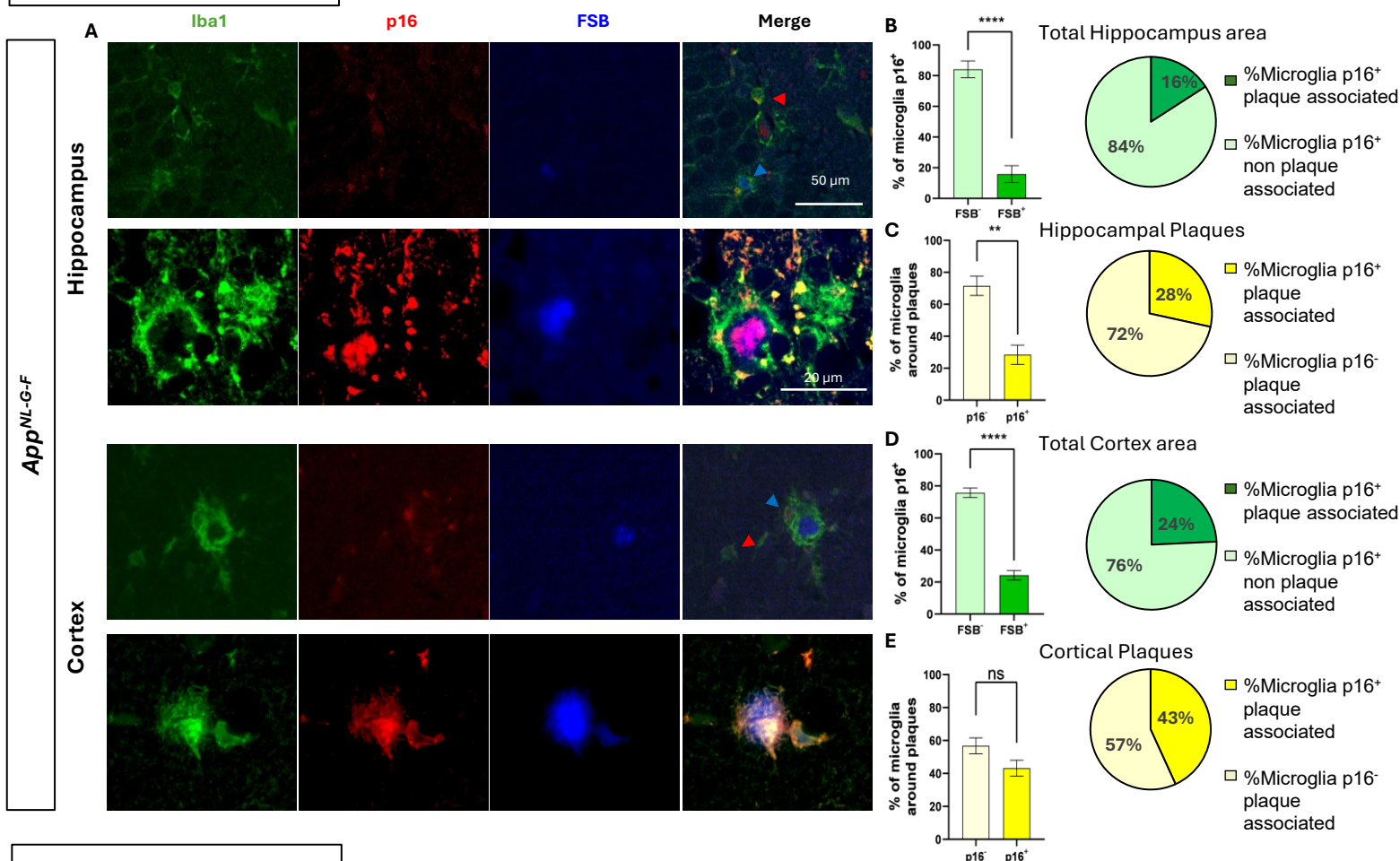

24-months-old

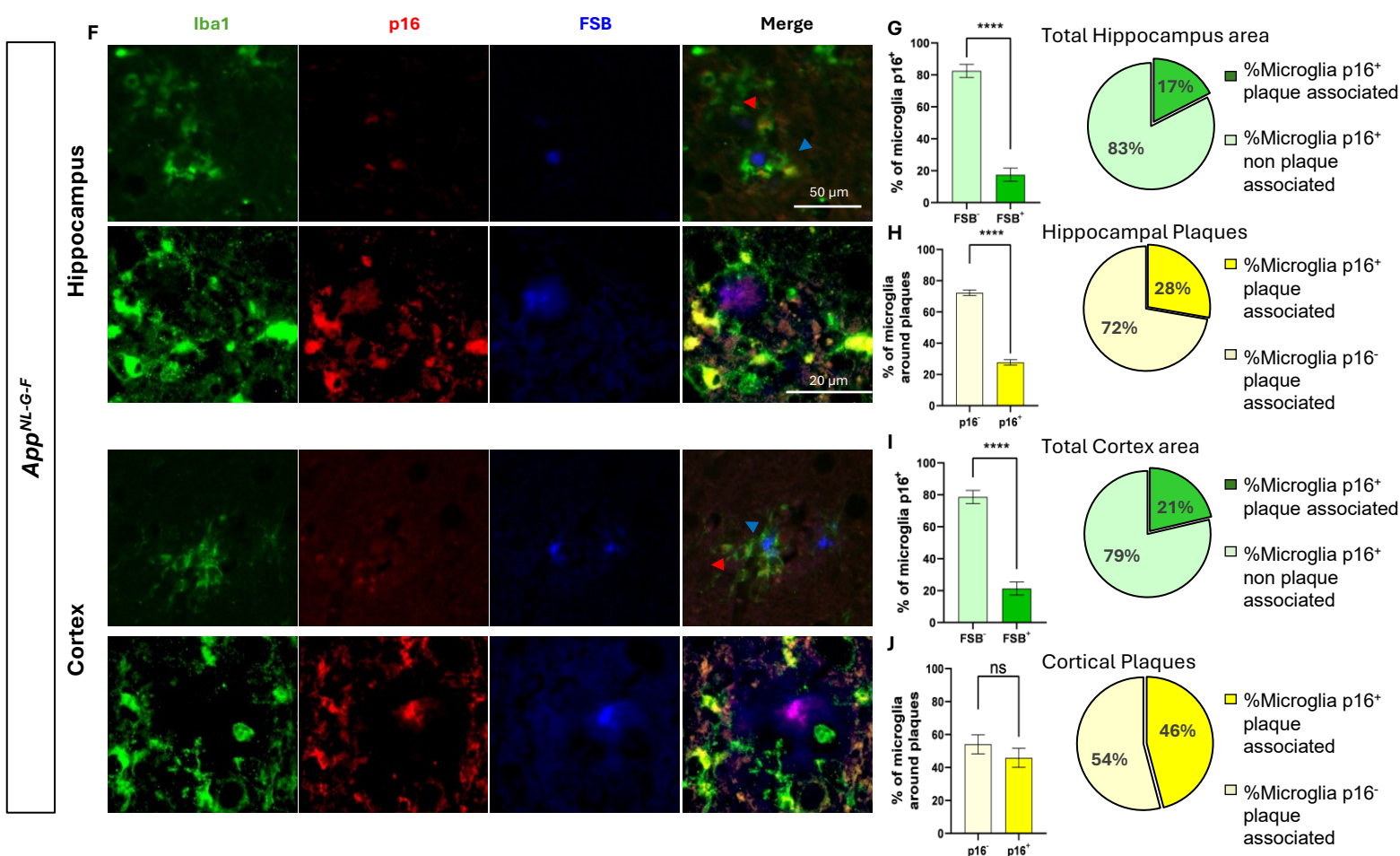

Supplement: Supplementary file 1 — Figure S1: Age‐Dependent Changes Grouped by Cell Type and Genotype, and SA‐βGal+Iba1 staining. (A) The percentage of positive cells has been averaged across all three Markers (p16, p21, and p53) and both Regions (Hippocampus and Cortex). This visualization clearly highlights the Genotype and Age effect on the three primary cell types. Microglia—and Astrocytes show a dramatic change in color from 4 to 12 months, confirming the accelerated pathology in these glial cell types that is effect of the genotype. The neurons WT row is showing a relatively high percentage of senescent markers already at 4 months and stays high in older ages, reinforcing the idea that baseline neural ‐marker expression is high regardless of the specific disease model, making the glial changes a much clearer finding. (B) Double staining of SA‐β‐gal+Iba1 showed a partial colocalization of SA‐β‐gal and microglia cells. Scale bars: 50 μm. Figure S2: Microglial proliferation and Skeletal analysis of microglia in WT and App NL‐G‐F mice. Quantification of total microglia (Iba1+) in the hippocampus (A) and cortex (B) of WT and App NL‐G‐F mice at 4, 12, and 24 months of age. Data are expressed as total microglia per area. Data are shown as mean ± SEM. Statistical analysis: one‐way ANOVA. **p < 0.01; ****p < 0.0001 versus WT group. Quantification of the number of microglial endpoints per cell and process length per cell in the hippocampus and cortex of 12‐month‐old WT and App NL‐G‐F mice. Images were acquired at 20× magnification and analyzed using ImageJ software. Statistical analysis was performed using unpaired t‐tests. Data are presented as mean ± SEM. *p < 0.05 versus WT group. Figure S3: Association of p16+ microglia with amyloid plaques at 12 and 24 months of age. The number of p16+ microglia and microglia surrounding amyloid plaques was assessed in both the hippocampus and cortex. At 12 months of age (A), data showed that 28% of the microglia surrounding plaques in the hippocampus (C) and 43% in [file ACEL-25-e70478-s001.pdf]
