# Supplementary material for: Cell Type‐Specific Expression of p16, p21, and p53 Reveals Age‐Dependent Glial Senescence in the AppNL‐G‐F Mouse Model of Alzheimer's Disease
Source: Aging Cell. 2026 Apr 14;25(4):e70478. doi: 10.1111/acel.70478 (PMC13078136; doi:10.1111/acel.70478)
Supplement: Supplementary file 2 — Table S1: List of the antibodies used in the study. [file ACEL-25-e70478-s002.docx]

**Supplementary Table 1**

List of the antibodies used in the study.

| **Antibody** | **Host and type** | **Dilution for IF** | **Product code** | **Source** |
| --- | --- | --- | --- | --- |
| p21 | Mouse monoclonal | 1:100 | sc-817 | Santa Cruz Biotechnology |
| p16 | Rabbit  monoclonal | 1:100 | ab211542 | Abcam |
| p53 | Mouse  monoclonal | 1:100 | sc-99 | Santa Cruz Biotechnology |
| Iba1 | Mouse  monoclonal | 1:200 | MA5-27726 | Invitrogen |
| Iba1 | Rabbit monoclonal | 1:500 | 019-19741 | FUJIFILM |
| NeuN | Mouse  monoclonal | 1:100 | MAB377 | Sigma |
| NeuN | Rabbit monoclonal | 1:200 | 26975-1-AP | Proteintech |
| GFAP | Mouse  monoclonal | 1:150 | 3670 | Cell Signaling |
| GFAP | Rabbit monoclonal | 1:500 | ZO334 | Dako |
| CD68 | Rat monoclonal | 1:200 | MCA1957GA | BioRad |
| Alexa Fluor 488  Anti-rabbit IgG | Goat | 1:500 | A32731 | Invitrogen |
| Alexa Fluor 546  Anti-mouse IgG | Goat | 1:500 | A-11003 | Invitrogen |
