# Supplementary material for: Cell Type‐Specific Expression of p16, p21, and p53 Reveals Age‐Dependent Glial Senescence in the AppNL‐G‐F Mouse Model of Alzheimer's Disease
Source: Aging Cell. 2026 Apr 14;25(4):e70478. doi: 10.1111/acel.70478 (PMC13078136; doi:10.1111/acel.70478)
Supplement: Supplementary file 3 — Table S2: Percentage of neurons, microglia and astrocytes positive for p16, p21 and p53 in WT and App NL‐G‐F mice at 4, 12 and 24 months of age. In the table are reported the percentage mean ± SEM. [file ACEL-25-e70478-s003.docx]

**Supplementary Table 2**

Percentage of neurons, microglia and astrocytes positive for p16, p21 and p53 in WT and *App^NL-G-F^* mice at 4, 12 and 24 months of age. In the table are reported the percentage mean ± SEM.

|  |  |  | Hippocampus | | |  | Cortex | | |
| --- | --- | --- | --- | --- | --- | --- | --- | --- | --- |
|  |  |  | % of positive cells | | |  | % of positive cells | | |
|  | Months | Genotype | **Neurons** | **Microglia** | **Astrocytes** |  | **Neurons** | **Microglia** | **Astrocytes** |
| p16 | 4 | WT | *4,8 ± 1,5* | *4,1 ± 2,9* | *18,6 ± 2,6* |  | *5,6 ± 1,1* | *4,1 ± 0,8* | *16,0 ± 8,6* |
|  |  | *App^NL-G-F^* | *4,5 ± 0,6* | *3,2 ± 2,2* | *13,5 ± 3,6* |  | *3,6 ± 1,2* | *5,0 ± 1,2* | *11,6 ± 1,9* |
|  | 12 | WT | 0,1 *± 0,1* | 11,0*±0,9* | 0,8 *± 0,3* |  | *0,9 ± 0,4* | 16,3 *±2,2* | 0,9 *±0,9* |
|  |  | *App^NL-G-F^* | 0,1 *± 0,1* | 25,8*± 3,6* | 2,5 *± 0,4* |  | 1,5 *± 0,5* | 30,8 *±1,5* | *4,7 ±1,0* |
|  | 24 | WT | 4,0 *± 0,5* | 11,0 *±3,5* | 6,6 *± 2,7* |  | 8,0 *± 1,5* | 9,6 *±2,4* | 5,3*±2,8* |
|  |  | *App^NL-G-F^* | 4,2 *± 0,1* | 37,0 *±2,1* | 6,3 *± 1,2* |  | 8,5 *±1,0* | 38,8 *±1,4* | 8,1 *±1,3* |
|  |  |  |  |  |  |  |  |  |  |
| p21 | 4 | WT | 31,7*±0,2* | 2,8*±1,6* | 55,3*±6,4* |  | 13,2*±4,4* | 1,5*±0,9* | 47,6*±2,4* |
|  |  | *App^NL-G-F^* | 29,8*±1,1* | 5,1*±1,5* | 50,2*±3,7* |  | 14,7*±3,2* | 6,0*±1,1* | 43,5*±5,7* |
|  | 12 | WT | 36,0*±4,7* | 2,4*±0,9* | 40,0*±3,3* |  | 28,6*±6,9* | 5,6*±0,4* | 26,9*±4,30* |
|  |  | *App^NL-G-F^* | 41,3*±2,4* | 36,13,8 | 85,8*±1,3* |  | 37,1*±3,0* | 33,4*±2,7* | 76,0*±1,3* |
|  | 24 | WT | 55,6*±5,0* | 23,8*±2,8* | 58,8*±5,6* |  | 44,1*±7,9* | 27,4*±1,3* | 55,7*±3,6* |
|  |  | *App^NL-G-F^* | 60,3*±1,0* | 48,8*±1,8* | 77,9*±3,5* |  | 52,9*±3,0* | 52,7*±*4,4 | 73,5*±3,4* |
|  |  |  |  |  |  |  |  |  |  |
| p53 | 4 | WT | 44,9*±3,4* | 1,3*±0,9* | 34,9*±4,5* |  | 26,3*±11,9* | 0,8*±0,8* | 26,9*±12,4* |
|  |  | *App^NL-G-F^* | 52,9*±5,1* | 2,7*±1,8* | 31,0*±5,1* |  | 17,7*±7,6* | 1,1*±0,6* | 28,5*±12,7* |
|  | 12 | WT | 57,6*±7,3* | *7,8±2,1* | 16,9*±4,2* |  | 46,9*±6,3* | 14,2*±2,7* | *12,6±5,7* |
|  |  | *App^NL-G-F^* | 69,3*±6,9* | *13,8±2,1* | 33,8*±5,3* |  | 57,0*±7,2* | 14,6*±1,6* | 23,3*±4,1* |
|  | 24 | WT | 52,9*±2,5* | 21,4*±4,0* | 66,4*±6,9* |  | 50,4*±3,9* | 23,3*±4,6* | 57,6*±6,9* |
|  |  | *App^NL-G-F^* | 48,0*±1,6* | 33,3*±2,3* | 60,6*±3,5* |  | 43,9*±4,1* | 40,9*±2,0* | 52,7*±4,4* |
